# Serotyping dengue virus with isothermal amplification and a portable sequencer

**DOI:** 10.1038/s41598-017-03734-5

**Published:** 2017-06-14

**Authors:** Junya Yamagishi, Lucky R. Runtuwene, Kyoko Hayashida, Arthur E. Mongan, Lan Anh Nguyen Thi, Linh Nguyen Thuy, Cam Nguyen Nhat, Kriengsak Limkittikul, Chukiat Sirivichayakul, Nuankanya Sathirapongsasuti, Martin Frith, Wojciech Makalowski, Yuki Eshita, Sumio Sugano, Yutaka Suzuki

**Affiliations:** 1 Division of Collaboration and Education, Research Center for Zoonosis Control, Hokkaido University, North 20 West 10, Kita-ku, Sapporo, Hokkaido 001-0020 Japan; 2 Global Station for Zoonosis Control, GI-CoRE, Hokkaido University, North 20 West 10, Kita-ku, Sapporo, Hokkaido 001-0020 Japan; 3 Department of Computational Biology and Medical Sciences, Graduate School of Frontier Sciences, The University of Tokyo, 5-1-5 Kashiwanoha, Kashiwa, Chiba 277-8562 Japan; 4 Oita University, Division of Laboratory Animal Science, Research Promotion Institute, 1-1 Idaigaoka, Hazamacho, Yufushi, Oita 879-5593 Japan; 5 Department of Clinical Pathology, Sam Ratulangi University, Kampus Unsrat, Bahu Manado 95115 Indonesia; 6 National Institute of Hygiene and Epidemiology, 1 Yersin, Hanoi, 112800 Vietnam; 7 Preventive Medicine Center Hanoi, 70 Nguyen Chi Thanh Street, 117257 Hanoi, Vietnam; 8 Department of Tropical Pediatrics, Faculty of Tropical Medicine, Mahidol University, Bangkok, 10300 Thailand; 9 Graduate Program in Translational Medicine, Research Center, Faculty of Medicine Ramathibodi Hospital, Mahidol University, Bangkok, 10300 Thailand; 10 Computational Biology Research Center, National Institute of Advanced Industrial Science and Technology, Tokyo Waterfront Bio-IT Research Building, 2-4-7 Aomi, Koto-ku, Tokyo 135-0064 Japan; 11 Faculty of Medicine, Institute of Bioinformatics, University of Münster, Niels-Stensen Strasse 14, Münster, 48149 Germany; 12 Department of Medical Entomology, Faculty of Tropical Medicine, Mahidol University, 420/6 Ratchawithi Road, Thung Phaya Thai, Ratchathewi, Bangkok 10400 Thailand

## Abstract

The recent development of a nanopore-type portable DNA sequencer has changed the way we think about DNA sequencing. We can perform sequencing directly in the field, where we collect the samples. Here, we report the development of a novel method to detect and genotype tropical disease pathogens, using dengue fever as a model. By combining the sequencer with isothermal amplification that only requires a water bath, we were able to amplify and sequence target viral genomes with ease. Starting from a serum sample, the entire procedure could be finished in a single day. The analysis of blood samples collected from 141 Indonesian patients demonstrated that this method enables the clinical identification and serotyping of the dengue virus with high sensitivity and specificity. The overall successful detection rate was 79%, and a total of 58 SNVs were detected. Similar analyses were conducted on 80 Vietnamese and 12 Thai samples with similar performance. Based on the obtained sequence information, we demonstrated that this approach is able to produce indispensable information for etiologically analyzing annual or regional diversifications of the pathogens.

## Introduction

DNA sequencing is a decisive diagnostic technique to identify pathogenic species, where the precise sequence provides further information on the pathogen genotypes, such as serotypes or single-nucleotide variations (SNVs). Despite its potential power, sequencing analysis is rarely used as a first diagnostic test in the medical field due to its technical and financial drawbacks. A recently available portable sequencer, MinION, is expected to drastically change the availability of sequencing analysis^1, 2^. Unlike conventional sequencing platforms, the MinION device determines base sequences by detecting changes in the ionic current as a DNA strand passes through a nanoscale ion channel^3^. Due to its lack of requirement to detect fluorescence, it does not need any optical device for signal detection, which reduces the size and cost of the sequencing instrument. MinION is a portable 90-gram DNA sequencing device that connects to a laptop computer through a USB cable and, along with its reagents, is reasonably priced. Converting a sample to a sequencing library can be completed in 2 hours and is simple, consisting of adaptor ligation to a viral amplicon generated under isothermal conditions, thus eliminating the need for conventional laboratory equipment and an electric supply. These features provide a great advantage for the precise genotyping of tropical disease pathogens in rural hospitals and in the field. In fact, very recently, several reports describing its first application for sequencing pathogens have appeared^4–6^.

For further simplifying the procedure, we utilized the loop-mediated isothermal amplification (LAMP) method for the sample preparation^7, 8^. LAMP is a simple method that amplifies DNA or RNA in a single isothermal reaction step. For the LAMP process, crude samples, such as serum samples collected from patients, can be used without any prior purification. Previous studies have demonstrated that the amplification is robust and occasionally even more sensitive than conventional RT-PCR methods^9–11^. However, the high level of sensitivity may be compromised because LAMP is also known to be error-prone, producing a number of false positives^10, 12^. In many cases, performing a detailed genotyping analysis based on only the LAMP method is practically impossible due to its high rate of cross-reactivity between different genotypes.

Here, we developed a new method for the identification and serotyping of a tropical disease pathogen, using dengue fever as the model. The dengue virus is an RNA virus that causes dengue fever^13^. More than 250,000 cases were reported in 2010, mainly in Southeast Asian countries^14^. The dengue virus has four known serotypes (DENV1-DENV4), which differ by 50–70% in their sequence identities within their approximately 11 kb single-stranded RNA genomes^15^. Different serotypes may result in different clinical manifestations^16, 17^, and a second infection by another serotype increases the risk of developing severe dengue^18, 19^. In addition, the observed symptoms of dengue fever are occasionally unique, even between cases with the same serotype^20^. These distinct features should be explained by changes in their genomic SNVs^21^.

In this study, we used the LAMP method to amplify the genomes of dengue viruses from patient blood samples. We amplified parts of the 3′UTR of the virus, as opposed to a previously published method^22^. We then identified their sequences with the MinION sequencer. The entire reaction process, starting from the serum sample, amplifying the viral genome, generating the raw sequence data and converting the obtained data into a readable sequence, can be completed in a single day, and none of the steps require the installation of large instruments. We further demonstrated the potential power of this approach by analyzing clinical samples from a total of 233 patients from Indonesia, Vietnam and Thailand. The combination of these pre-existing methods would be beneficial to clinical applications in the near future.

## Results

### Serotyping dengue viruses using laboratory materials

**Figure 1:**
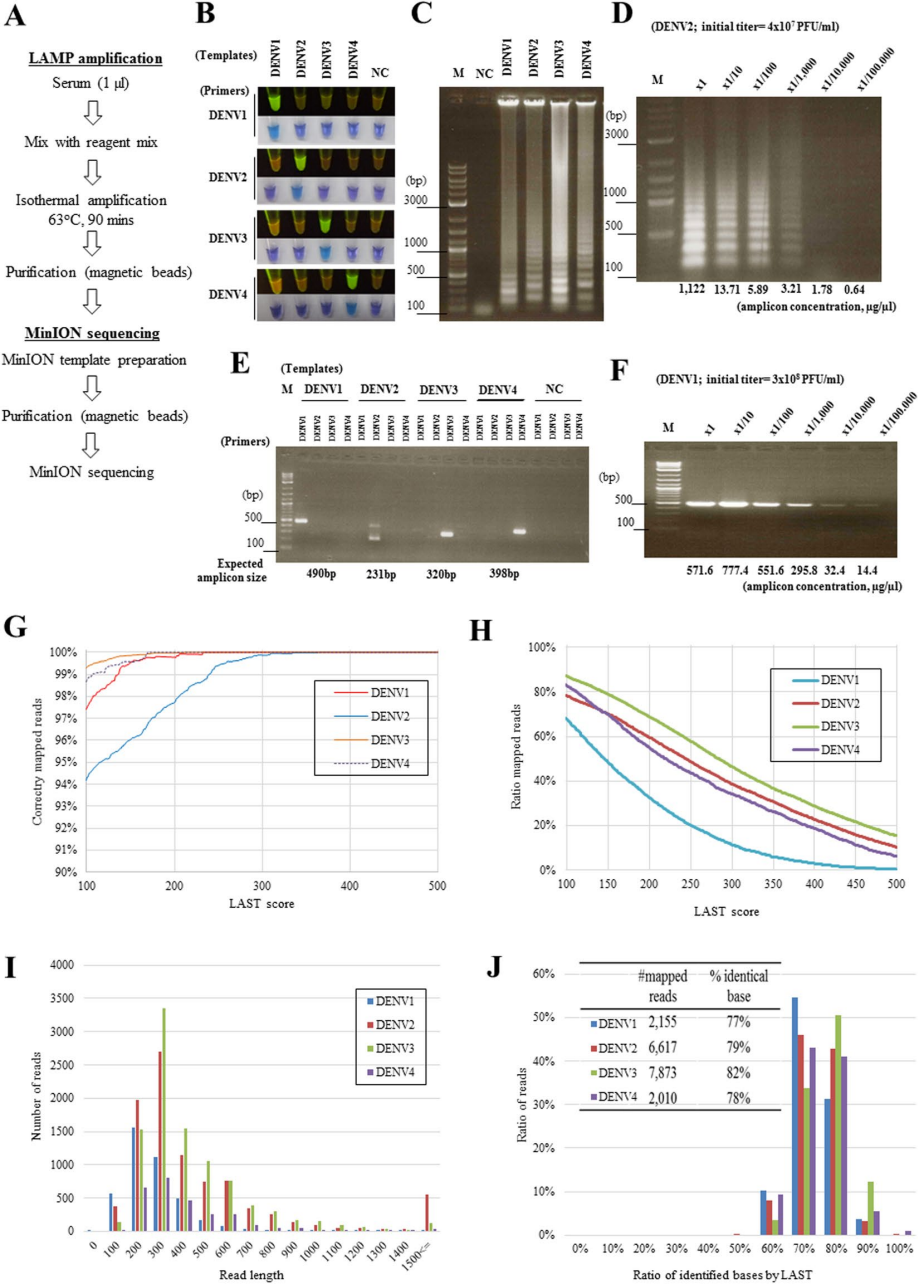
Serotyping analysis of dengue virus using LAMP and MinION. (**A**) Schematic representation of the method using the LAMP amplification and MinION sequencing. (**B**) UV and visual light images and (**C**) agarose gel electrophoresis image of the LAMP amplicons obtained from the control templates. For (**B**) the top rows for each serotype are the UV images, and the bottom rows are under visual light. (**D**) Electrophoresis image of the LAMP products generated from a serially diluted control template of DENV2. The amplicon concentration was calculated using the Agilent Bioanalyzer. (**E**) RT-PCR amplification of the control viruses. (**F**) RT-PCR analysis of the DENV1 serially diluted control template. The amplicon concentration was calculated by the Agilent Bioanalyzer. (**G**) Precision and (**H**) recall rates of the sequence reads recovered at the cut-off score of LAST indicated on the *x*-axis. (**I**) Length distribution of the aligned reads. (**J**) The accuracy of the sequencing obtained by the flow cell version 7 was calculated by considering the matched bases between each sequence and the corresponding reference genome. The images from (**B**) to (**F**) are representative of three biological replicates.

The overall scheme for the sample processing and sequencing analysis of a dengue virus is shown in Fig. 1A (see also Supplementary Fig. S1a). Briefly, the LAMP amplification using the serotype-specific primers is carried out by mixing pre-mixed reagents, including a reverse transcriptase, directly with the serum. After 1 hour of incubation at 65 °C, the positive reaction product is purified by magnetic beads, which requires only a magnetic stand but no other specific equipment, such as a centrifuge. Next, the MinION template preparation is also a single-step reaction that ligates the sequencing adaptors and the motor protein to the template DNA. The resulting product is loaded into the MinION sequencer, where the sequencing reaction occurs for another 6–48 hours, depending on the setting. A detailed protocol is described in the Methods.

First, we attempted to examine whether the presented scheme worked correctly. For the purposes of optimizing the experimental conditions and validating the method, we used *in vitro* cultured dengue viruses from the corresponding serotypes (DENV1-DENV4) as templates (see Supplementary Fig. S1b for the amplification target and primer positions). We observed the correct amplification of the viral genomes by UV light examination, showing that LAMP successfully detects the corresponding serotypes in a highly selective manner on its own (Fig. 1B). These results were further confirmed by observing the expected tandem amplification of the products with agarose gel electrophoresis (Fig. 1C). A serial dilution analysis showed that the 10^−4^ dilution (estimated 4,000 pfu/ml), depending on the serotype, yielded approximately 100 ng of amplicon DNA, which is the current minimum requirement for a sequencing reaction (Fig. 1D). We compared these results with those of the serotype-specific RT-PCR followed by Sanger sequencing (Fig. 1E and F; also, see Supplementary Fig. S1c) to identify the specificity and the sensitivity. Consistent with results from previous reports, we confirmed that both methods identified and distinguished between the respective serotypes of the control templates in a laboratory setting^23^.

The obtained LAMP products were used for the MinION template preparation and then for the sequencing analysis. We used an entire MinION flow cell per sample and obtained a total of 5,000–20,000 reads (2,500–10,000 reads when only “2D” reads, which are consensus sequences of the reads on both strands, were counted), depending on the run (Supplementary Table S1a). The sequences were mapped to the reference genomes of the serotypes (DENV1-DENV4) using an alignment program, LAST^24, 25^, with varying threshold mapping scores and other parameters. For example, as shown in Fig. 1G and H, reasonable precision and recall rates were obtained with a mapping score of 150 and a mismap score of 1 (which is approximately equivalent to e = 5e-5, see Supplementary Fig. S2). Further details on the results using various analysis programs and the LAST score are shown in Supplementary Table S1b and c. Under these settings, the alignments matched the reference genomes of the serotypes used for the input in an almost perfectly exclusive manner (Table 1). We also found that, even though the values decreased when the primer sites were completely masked, the results were still significantly different between serotypes (Supplementary Table S2). Since the inception of MinION, it has been improved for accuracy and ease of operation. The newly updated version of the flow cell has a better sequencing accuracy compared to the old version that we used for the entirety of this work (Supplementary Fig. S3, Supplementary Table S3). For this and the subsequent sequencing runs, only “2D” reads were used for the analysis.

**Table 1:** Sequence statistics.

Sample ID	Sequencing mode	Total reads	Total mapped reads (score = 150)	Total reads mapped to the corresponding serotype (score = 150, mismap = 1)				Ratio
				DENV1	DENV2	DENV3	DENV4	
Control DENV1	2D	4,060	2,164	2,155	0	6	3	100%
Control DENV2	2D	9,260	6,719	11	6,617	12	79	98%
Control DENV3	2D	9,750	7,885	11	1	7,873	0	100%
Control DENV4	2D	2,758	2,014	0	0	4	2,010	100%

Sequence data statistics from the control templates of *in vitro* cultured viruses showing the number and percentage of sequence reads that correspond to the genome sequences of the indicated serotype for each control sample.

Based on the alignment, we calculated the length of the matching sequence reads (Fig. 1I). We found that most of the reads were less than 500 bases, but some longer reads were also obtained, reflecting the presence of multiple LAMP tandem amplicons within a single read. Although it is possible to use all the tandem sequences for further evaluation, we used the best matched sequence from a single read for the following analysis. Then, we examined the sequencing fidelity (Fig. 1J). We found that the overall sequence identity was 80%, with the remaining 20% including 12, 3 and 6% with base substitutions, insertions and deletions, respectively, showing that there is no particular, if any, bias in a base-substitution pattern at this level (Supplementary Table S4a and b). We further constructed a consensus sequence for each sample, putting the aligned sequence information together (see Methods for details), and then compared the constructed consensus sequence with the reference. We found that, despite the low sequence quality in a single read, the sequence identities between the consensus sequences and the reference genomes were 97%, on average (including the sites that turned out to be SNVs; see below). Together, these results demonstrated that the identification and serotyping of the dengue virus is possible using the scheme shown in Fig. 1A.

### Detection and serotyping of dengue viruses for clinical samples

**Figure 2:**
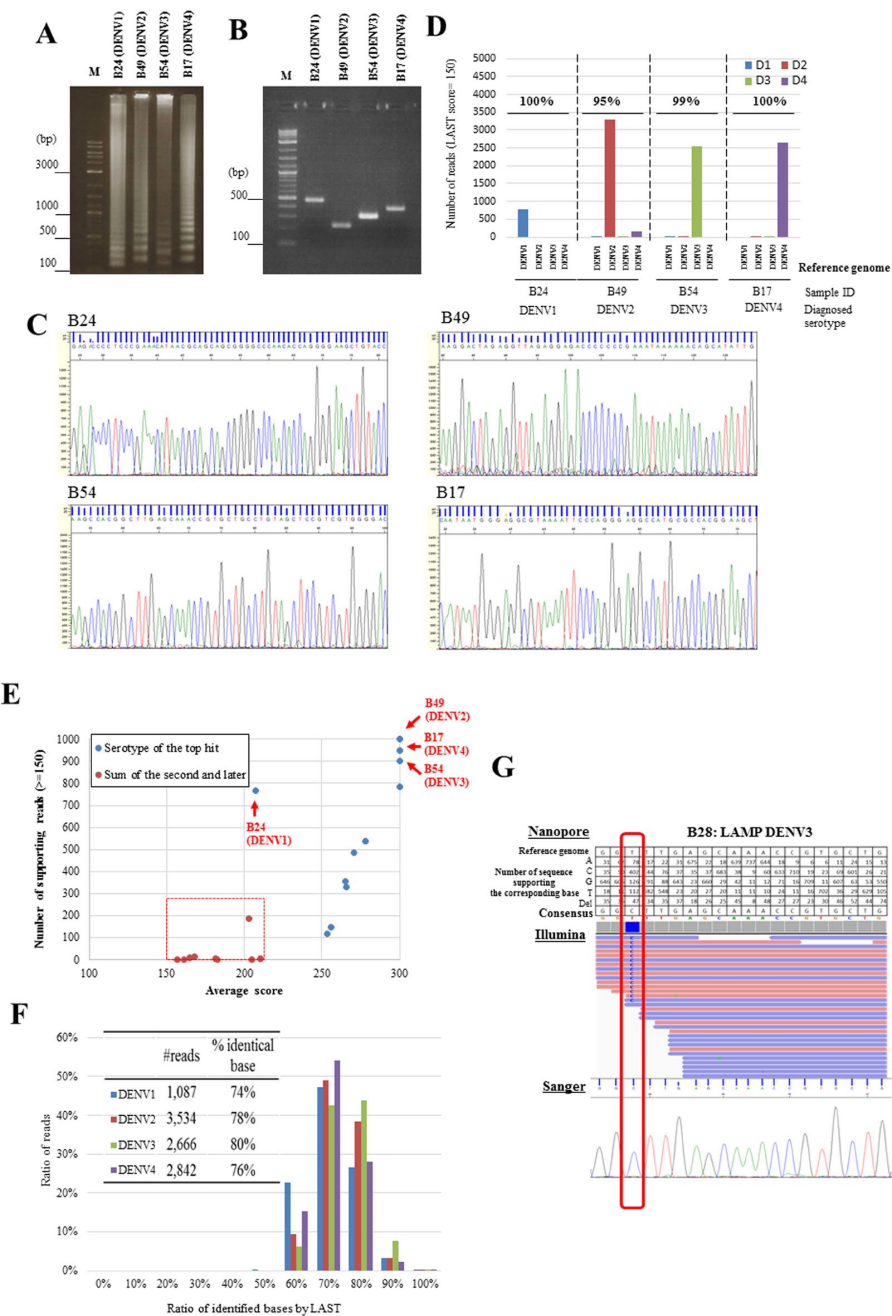
Serotype detection of clinical samples. (**A**–**C**) Results of the LAMP analysis (**A**), RT-PCR analysis (**B**) and Sanger sequencing (**C**) using clinical samples (n = 4). (**D**) Results of the MinION sequencing; the numbers of sequence reads corresponding to the indicated serotypes are shown. (**E**) Result of alignment for each sample, plotted according to the number (*y*-axis) and the average LAST scores of the reads that supported the serotypes of the top hits (blue dots) and the later hits (red dots) (n = 11). The range of negative signals (red dots) is indicated by the broken red box. Note that its range is clearly separate from the positive signals (blue dots). (**F**) The accuracy of the sequencing as calculated above. (**G**) Upper panel, MinION sequence alignment between the generated consensus sequence from a particular patient and the reference genome. Middle and lower panels, results from Illumina (middle panel) and Sanger sequencing (lower panel) using the same sample. The position of the identified SNV is highlighted by the red box. This image is a representation of one patient sample.

The developed scheme was used to determine the serotypes of clinical samples, specifically serum collected from Indonesian patients who were suspected to have dengue fever (all of the information is summarized in Additional File 1). For each sample, 1 µl of the serum was used for the LAMP amplification (Fig. 2A). Only a small number of cases were positive for serotype-specific LAMP reactions, which was not observed for the control samples. In most cases, seemingly ambiguous signals were detected from more than one serotype-specific primer set, possibly due to the cross-detection of non-serotype-specific amplifications. Even in the “healthy” control cases, LAMP occasionally detected erroneous signals (see Supplementary Fig. S4a for example), which were then verified by MinION (Supplementary Table S5a). This emphasized the necessity for further sequencing verification after the LAMP analysis.

For the simplest case, we first selected four samples in which the clearest signals were detected for each of the serotypes, DENV1 to DENV4. For these samples, we conducted the conventional RT-PCR validation and found that the results were consistent with those of the LAMP analysis (Fig. 2B). A Sanger sequencing analysis of the RT-PCR products further confirmed that the corresponding serotypes were accurately detected (Fig. 2C). For these samples, the LAMP amplicons were subjected to MinION sequencing, similarly to the cases of the *in vitro* samples (Table 2a). Again, MinION was able to detect and distinguish between serotypes, demonstrating that the developed method also worked with clinical samples (Fig. 2D). We then randomly chose another seven cases that did not always show clear LAMP results for only one serotype. For these samples, we mixed the LAMP positive amplicons for the MinION sequencing and found that the same parameters used for the above samples clearly separated the serotypes (Fig. 2E; Table 2b). Verification by RT-PCR and Sanger sequencing showed that the results were consistent for all of the cases in which RT-PCR gave positive results. It should also be noted that only our method detected and distinguished the serotypes in cases where RT-PCR failed in the detection of the virus genome (Supplementary Table S5b and c).

**Table 2:** Serotyping clinical samples.

Sample ID	Sequencing mode	Category	Diagnosed serotype	Total reads	Number of mapped reads	Number of reads mapped to the corresponding serotype	Percent ratio	Average sequence identity
**a**								
B24	2D	Clinical representative	DENV1	3,156	1,091	1,087	100%	74%
B49	2D	Clinical representative	DENV2	4,680	3,610	3,534	98%	78%
B54	2D	Clinical representative	DENV3	5,598	2,677	2,666	100%	80%
B17	2D	Clinical representative	DENV4	4,220	2,846	2,842	100%	76%
**b**								
01	2D	Clinical	DENV1	243	132	130	98%	77%
11	2D	Clinical	DENV1	867	525	522	99%	77%
17	2D	Clinical	DENV1	600	382	381	100%	77%
21	2D	Clinical	DENV1	255	159	159	100%	76%
B03	2D	Clinical	DENV1	609	354	351	99%	77%
B25	2D	Clinical	DENV1	832	563	563	100%	79%
B28	2D	Clinical	DENV3	986	808	806	100%	81%

The number and percentage of the sequence reads supporting the indicated serotypes are shown in the seventh and eighth columns, respectively, for the indicated clinical samples. The average sequence identification was calculated based on the alignments and is shown in the last column. Shown here are results from the representative samples for DENV1 to DENV4 (**a**) and random selection (**b**).

We also evaluated the overall sequence fidelity for these eleven cases (four clinical representatives with clear LAMP results and seven other clinical samples). Similar to the cases in *in vitro* samples, the sequence fidelity in each read was 77%, on average (Fig. 2F). However, the sequences with the highest score had a fidelity of >95% (Supplementary Fig. S4b). Other sequencing features, such as the sequence identity and mismatch patterns, resembled the cases in the control samples (compare Supplementary Table S5d to Supplementary Table S4a). When we generated a consensus sequence using all the reads together, an average fidelity of 98% was obtained. We further examined the possibility that some of the remaining 2% mismatch sites may represent SNV sites. We selected the mismatch sites that were supported by a sufficient number of MinION sequences, whereas the reference-type base was supported by fewer sequences (see Methods for details). We found a total of 7 such sites in 6 samples (see Supplementary Table S5e). On these sites, we conducted Illumina sequencing and found that 6 were confirmed as SNVs by Illumina sequencing and Sanger sequencing (see Fig. 2G for an example).

Finally, we used the entire procedure in field conditions, where no laboratory equipment was available. The serum was amplified by LAMP in a water bath, and the resulting amplicons were used for sequencing without any quantification. MinION sequencing was conducted with a laptop computer as the platform. The entire procedure was performed at a local small clinic in Indonesia with a resident medical doctor (Supplementary Fig. S5) using locally prepared chemical reagents. Even in these circumstances, we successfully identified the viral genome and separated its serotypes. Notably, the results from the part of the samples that were analyzed solely in the field were almost identical to those from laboratory analyses (Supplementary Table S5f). These results further demonstrated that this novel procedure is a convenient and robust method that is well-suited for field applications.

### Scaling the MinION assays by introducing barcoding sequences

To further scale the analysis, we attempted to enable the analysis of multiple samples on a single MinION sequencer. First, to determine the possible extent of multiplexing, we estimated the sequence depth necessary for correct serotyping. We serially split the obtained sequence reads from a single run and simulated how many total reads should sufficiently represent significant information for detecting the viral genome and identifying its serotype. This analysis indicated that a total reads of >50 would yield counts of >10 reads clearly supporting a particular serotype for the majority of the samples (Supplementary Fig. S6a). To obtain such reads for each sample, a MinION flow cell may have the potential for analyzing at least ten different samples (inset of Supplementary Fig. S6a). For designing barcodes, because of the low sequence fidelity of the MinION sequencer, we allocated 24 bases for the barcoding oligo (within the F1C sites of the corresponding serotypes; see Supplementary Table S6 for their sequences), and we evaluated how precisely a particular barcoding sequence could be separated from the others at a given sequencing fidelity and depth. We found that at a LAST score of 100, the barcoding sequences could be separated in a reasonably accurate manner (precision and recall rates being 0.87 and 0.61, respectively; Supplementary Fig. S6b and c). Utilizing the designed barcoding oligos, we mixed nine samples with different barcode oligos and sequenced them on a single MinION sequencer. An average of 37% of the reads were discarded as unresolved barcoding sequences, which is within the expected range. Nevertheless, a sufficient number of sequence reads were obtained for each sample (see Supplementary Table S7a).

### Clinical applications for Indonesian, Vietnam, and Thailand samples

**Figure 3:**
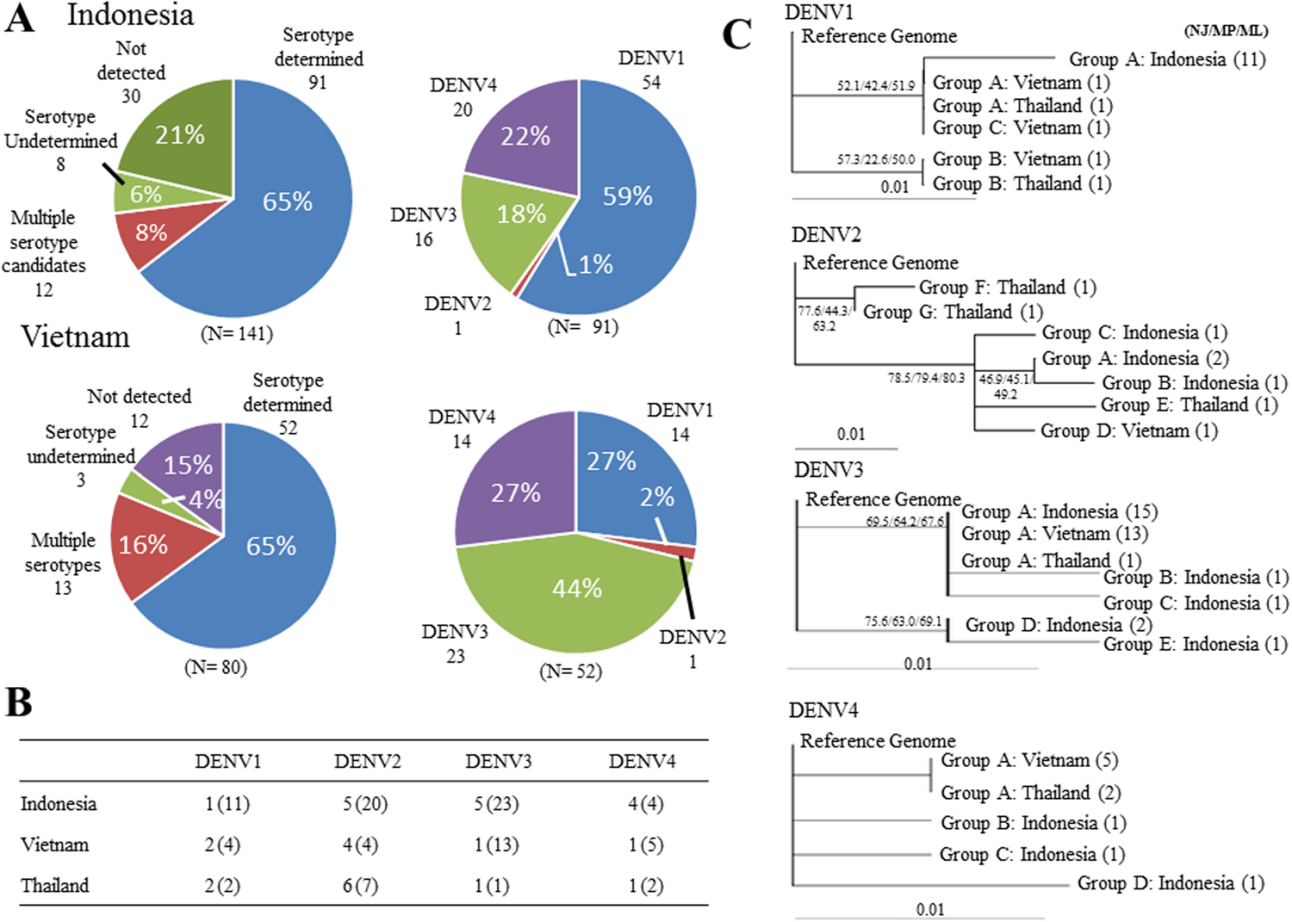
Serotyping of samples from Indonesia, Vietnam and Thailand. (**A**) Field application results from the serotyping analysis of 141 and 80 dengue patients from Indonesia and Vietnam, respectively. The left panels show the results of the viral identifications and their serotyping, and the right panels show the composition of the serotypes in this population. (**B**) SNVs found in each country for each serotype. The numbers in parentheses show the numbers of all samples that have SNVs, while the numbers outside the parentheses show the unique SNVs among those samples. (**C**) Phylogenetic tree showing the relationship of the DENVs from Indonesia, Vietnam and Thailand to the reference DENV for each serotype. A genetic distance of 0.01 is represented by the horizontal bar. Cross-validations by NJ (neighbor-joining)/MP (maximum parsimony)/ML (maximum likelihood) methods are shown in the margin. See the Materials and Methods section for further details. Note that for (**B** and **C**), the SNVs detected from the “serotype determined” and “multiple serotype candidates” groups were used.

Putting the developed pieces of the procedures together, we analyzed 141 samples from Indonesian patients. Of these, 30 samples did not produce a LAMP amplicon. For the remaining 111 (79%) samples, we conducted MinION sequencing. We detected the viral genome in 103 (73%) samples. Of these, clear serotype detection was possible for 91 (65%) cases. Indeed, based on this analysis, we found that the DENV1 serotype was dominant in this population (Fig. 3A, upper). To validate these results, we conducted Illumina shotgun sequencing of the LAMP amplicons. We compared the results of the MinION and Illumina sequencing for 52 samples and found that the results were consistent in 49 of them. For the remaining 3 cases, Illumina detected only complex amplicons, which seemed to be errors generated from the LAMP amplifications. RT-PCR validation also verified ten out of the eleven serotypes determined by MinION (Supplementary Table S5g). Again, our method could detect the viral genome where the RT-PCR result was negative using the same amount of starting material. Additionally, we generated a consensus sequence for each case to identify SNVs and detected a total of 15 possible SNV positions in 37 samples (a total of 58 SNVs; Fig. 3B, Supplementary Table S7b). We then examined whether those SNVs could be verified by Illumina sequencing and found that most of the sites examined overlapped the SNV sites detected by Illumina (78% of the SNPs were sequenced and directly confirmed).

To further improve the sequencing analysis in a clinical setting, we compared the MinION performance in the present study with that in a recently published study^26^, as there is a concern that our performance is somewhat lower in term of the sequencing identity, particularly for clinical samples. We conducted the methods mentioned, but we did not see any alignment improvement. We, however, noted that many of the previous MinION evaluations counted only substitutions, while we also counted insertions and deletions. When we removed insertions and deletions from the analysis, we found that our performance is almost equal to previous results (Supplementary Table S8). We also plotted the precision against recall to evaluate the parameter dependency of the precise SNV detection using the Illumina sequencing as the correct dataset and found that we had employed a conservative threshold for detecting the SNVs. Our threshold of 2.0, although it has 100% precision, yields only 50% recall. We tried to change the threshold to 1.0, which would yield a greater than 70% recall, but the results were quite sensitive depending on the datasets, so we decided to remain with the conservative threshold (Supplementary Fig. S7).

Despite the generally successful sequencing analyses for the majority of the cases, we were concerned that there remained 12 (8%) “multiple-serotype” cases (note that we included those cases for the SNV detection, too) and 8 and 30 (6% and 21%) “serotype undetermined” and “not detected” cases, in which the serotypes of the viruses were vague or the virus genome itself was not detected, respectively. Further evaluation revealed that fewer reads and lower mapping rates were observed in these elusive cases (Supplementary Fig. S8a). The cause of the inadequate (and perhaps inaccurate) amplification was evaluated by investigating the laboratory evidence for the dengue fever diagnosis (Supplementary Table S9). Although we could not ignore the possibility of an amplification error by LAMP, we identified that the cause of the detection of multiple serotypes was likely contamination. To evaluate the degree of contamination, we serially mixed control viruses of the different serotypes and detected less than a 1/100 contamination of a very low viral titer (less than 10 p.f.u.; Supplementary Fig. S8b). Still, at least in some cases, there remains the possibility that patients may be doubly infected with viruses of different serotypes, considering the high sequence representations of both of the serotypes. Indeed, several such cases have been reported^27–30^. It would be intriguing if doubly infected cases occasionally appear, particularly in pandemic regions. Nevertheless, a better instruction to prevent future contamination should be implemented.

We attempted to demonstrate that the developed approach would facilitate a large-scale data production for serotyping dengue virus worldwide. For this purpose, we conducted similar studies in Vietnam and Thailand, where the circumstances are significantly different. The forms of the available samples differed depending on the region. In Vietnam, we collected 80 samples, either in RNA or serum (Additional File 2). In Thailand, three strains of immortalized primary culture viruses were available for each of the four serotypes (12 samples in total) instead of the primary viral samples (Additional File 3). We similarly conducted the LAMP-MinION sequencing analysis for a total of 92 samples. The results are mostly similar to those obtained from the Indonesian samples (Fig. 3A). For the Vietnamese samples, 65% of them were able to be identified as belonging to an individual serotype, and the remaining samples were either “multiple serotype candidates” (16%; cases where the serotypes could not be discriminated, mainly due to insufficient sequence fidelity), “serotype unknown” (4%; cases where the signals were too weak to discriminate the serotypes) or “not detected” (15%) (Fig. 3A, lower, left panel). For the Thai samples, successful and un-ambiguous serotyping (“serotype determined”) was achieved in eleven cases (92%), starting from culture media. We also compared the ability to identify the virus and distinguish it into different serotypes when the samples were prepared from RNAs or directly from serum. When the same samples were prepared from RNAs and serum, the results were mostly consistent with each other (Supplementary Fig. S8c). Upon closer inspection, using RNA as a starting material gave slightly better results than using serum (Supplementary Fig. S8d). However, RNA may not be an ideal material, as the isolation of RNA requires more manipulation than serum and more purification steps, making it more difficult to prepare on site. For the Vietnamese samples, real-time RT-PCR was conducted using the RNA samples. When we compared the Ct values, which represent the initial viral titer, we found that the samples having higher Ct values are less likely to be detected (success rates of 95% and 76% for the cases having Ct values of <27 and > = 32, respectively; Supplementary Fig. S8e and f), suggesting that the detection success rate depends on the initial viral titer.

For the “serotype determined” cases, we further analyzed the breakdown of the serotypes (Fig. 3A, lower, right panel). We found that the most frequently observed serotype was DENV3 (44%), followed by DENV1 (27%) and DENV4 (27%). The compositions of the serotypes determined are similar between the Indonesian and Vietnamese samples, and the fewer DENV2 samples is typical of these two countries, as well. For the Thai samples, we could detect the expected serotypes for eleven out of the twelve samples that were primarily cultured. The success detection rates were far higher than for the Indonesian and Vietnamese samples, due to their superior purity and higher viral titers (Additional File 3).

Based on the collected sequence information, we similarly called the SNVs for these samples, as well. Overall, 8 and 10 SNVs from 22 and 8 samples were identified from the Vietnam and Thailand studies, respectively (Fig. 3B, Supplementary Table S7c and d). We compared the detected SNVs among the Indonesian, Vietnamese and Thai samples to conduct a phylogenetic analysis of the dengue viruses in Southeast Asian countries (Fig. 3C). We found that each of the serotypes was roughly separated based on its originating regions, with a few exceptions where similar genetic variations were identified in geographically separated regions, which may reflect the recent endemic history of the viruses. Previous studies also demonstrated that several endemic histories could account for the observations obtained from the phylogenetic analyses (Supplementary Table S10), although we could not directly associate those results with the results of the present study, due to the difference in the target genomic regions. The current comparison and analyses are based on limited data, that is, the SNVs of the limited 3′UTR region of the viral genome and the samples of a total of 233 patients collected at arbitrary time points. To directly identify the functionally relevant SNVs, focusing on a particular genomic region may be needed. It is also worth noting that short amplicons have limited power to generate phylogenetic trees. Nevertheless, we included this analysis to demonstrate what MinION could achieve. Future extensive analyses will be required to reveal the correlation of the geography and annual changes to the viral genome using longer amplicons. Our presented method will provide a practical means to conduct an on-site sequencing diagnosis.

## Conclusion

In this study, we developed a convenient and precise method for the sequencing analysis of tropical disease pathogens. By combining LAMP amplification and the MinION sequencer, we have designed a method to identify and serotype DENV as a proof-of-concept. LAMP’s simplicity and MinION’s portability are two factors that allow the application of this method in the field in developing countries. We have shown that our method is able to differentiate the DENV serotype with good results, but MinION’s performance is rapidly improving. We compared older and newer versions of the flow cell and showed that the newer version yielded more sequencing reads and better accuracy (Supplementary Fig. S3 and Supplementary Table S3). It is also becoming more cost-efficient (see Supplementary Table S11 for details), which will further facilitate the wider application of this method. Our proof-of-concept works with DENV, which has a 50–70% difference in genome sequences among the serotypes. However, depending on the portion of the genome amplified, this method could also separate very closely related viruses. With the increasing number of clinical cases in which the genotypes of infecting pathogens are identified, progress in the development of knowledge of the etiology of tropical diseases will be accelerated. In that sense, we expect that this study will have a significant impact on the clinical practices in developing countries.

## Methods

### Samples and MinION sequencing

Control viruses were obtained from the National Institute of Infectious Disease, Japan (prototype virus of dengue 1^31^, Hawaiian strain; dengue 2, Trinidad 1751 strain (GenBank: EU073981)^32^; prototype virus of dengue 3, H-87 strain (GenBank: AB609590)^31^; prototype virus of dengue 4, H-241 strain (GenBank: KR011349)^31^). The clinical samples and corresponding clinical information were collected at Sam Ratulangi University in Manado, Sulawesi, Indonesia. All blood collection was carried out in accordance with relevant guidelines and regulations. Informed consent was obtained from all patients. All experimental protocols were approved by the Ethical Committee of Sam Ratulangi University.

The LAMP method was used to amplify the viruses as previously described^22, 33^ with a slight modification. The primers for serotyping have been previously published^34^, and we modified the primers to accommodate barcoding for MinION sequencing. Briefly, 1 µl of patient serum was added to the reaction solution, which contained 9.97 µl of 0.1% Triton X-100, 0.5 µl of 1 M Tris-HCl (pH 8.8), 0.25 µl of 1 M KCl, 1.75 µl of 100 mM MgSO_4_, 1.4 µl of 25 mM dNTPs (NipponGene), 1 µl of GelGreen 10,000x solution (Biotium), 0.1 µl of RNase inhibitor (Nacalai Tesque), 1 µl of Bst HS polymerase (New England Biolabs), 0.03 µl of AMV reverse transcriptase (NipponGene), and 2 µl of 2 M trehalose (Wako) at 63 °C. After 90 minutes of amplification, the amplicons were quantitated with a BioAnalyzer or evaluated by agarose gel electrophoresis. A Genomic DNA Sequencing kit (SQK-MAP-004) was used to prepare the library for MinION sequencing, following the manufacturer’s instructions. The LAMP amplicons were end-repaired using the NEBNext End Repair Module (New England Biolabs). The 100-µl reaction solution was incubated at room temperature for 30 minutes and purified using 100 µl of Agencourt Ampure XP beads (Beckman Coulter). The obtained products were used for dA-tailing with the NEBNext dA-tailing module (New England Biolabs) in a total volume of 30 µl at 37 °C for 30 minutes. Adaptor ligation was performed using reagents from Oxford Nanopore and the Blunt/TA Ligase Master Mix (New England Biolabs). The reactions were performed with dA-tailed DNA added to 10 µl of adapter mix, 2 µl of HP adapter, 8 µl of molecular grade water and 50 µl of Blunt/TA Ligase Master Mix at room temperature for 10 minutes. The adapter-ligated DNA was purified using 10 µl of Dynabeads His-Tag Isolation & Pulldown (Life Technologies) and washed with buffer from Oxford Nanopore. The samples were eluted in 25 µl of elution buffer from Oxford Nanopore.

During the sequencing, the MinION Flow Cell R7.0, R7.3, or R9.4 was used with the MinION device. Before sequencing, the flow cell was primed using 150 ml of EP buffer twice for 10 minutes. The sequencing library mix was prepared by combining 6 µl of the library with 140 µl of EP buffer and 4 µl of fuel mix. Then, the library mix was loaded onto the MinION Flow Cell. For the sequencing reaction, the genomic DNA 48-hour sequencing protocol was used with MinKNOW software. The base call was made with the Metrichor Agent (https://metrichor.com) using the workflow r7.X 2D Basecalling version 1.24. The base-called sequence data in fast5 format were converted to fastq format using Poretools^35^, and the fastq data were used for the subsequent analysis. RT-PCR was performed as described in Supplementary Method S1 for the validation analysis. The PCR primers and sequence primers used for the corresponding assays and the experimental procedure are shown in Supplementary Table S6. Sanger sequencing of the RT-PCR and LAMP products was conducted using a standard protocol.

### Computational procedure

The MinION read sequences are available in the DDBJ Sequence Read Archive (DRA) as accession number DRA004083. The sequences were mapped to the reference genomes of dengue viruses (NC_001477.1, NC_001474.2, NC_001475.2 and NC_002640.1 for DENV1, DENV2, DENV3 and DENV4, respectively). To map a sequence, the alignment program LAST (version 5.48) was used with the options ‘-r6 -q12 -a15 -b3 -e150 -m100 -Q1 -j4’ for the lastal command and ‘-m1’ for the last-split command. Note that with the recently improved version of last-train, we suggest replacing ‘-a15 -b3’ with ‘-a12 -A15 -b4’, which sets a lower cost for deletions than insertions. When necessary, the genomic sequences corresponding to the primer sites were masked. The number of mapped reads was counted at the indicated threshold. For instance, when detecting serotypes, any serotype supported by more than ten sequence tags corresponding to a LAST score >150 was considered. To separate the barcoding oligos, the sequences that matched multiple barcodes with a LAST score <100 were discarded. Based on the alignments, the most frequent base at each genomic coordinate was selected as a “consensus” for each sample. When the sequence depth did not exceed ten, the base was considered “N.” When calling SNVs were found, the generated consensus sequences were further compared with the reference genomes. An SNV was identified when the sequence reads supporting the variant were twice as numerous as the rest of the bases taken together. For the SNV calling based on Illumina sequencing, sequencing was conducted using a TruSeq DNA kit with the HiSeq2500 platform at the 36-base single-end. The sequences were aligned using BWA (http://bio-bwa.sourceforge.net/) and SNV calls that were made using GATK (https://software.broadinstitute.org/gatk/) with the default settings. Statistical significance was evaluated by the indicated methods using the statistical analysis software R (https://www.r-project.org/).

For the phylogenetic analysis, Muscle (ver. 3.6.31; http://www.ebi.ac.uk/Tools/msa/muscle) was used for generating sequence alignments. Further analysis was conducted using Phylip (ver. 3.696; http://evolution.genetics.washington.edu/phylip.html). Using the obtained sequence alignments, the phylogenetic tree was drawn by the maximum likelihood method using the Dnaml program in the Phylip package. For the bootstrap analysis, 1,000 bootstrap sequences were generated by the Seqboot program in the Phylip package. For the neighbor-joining method, distance matrix tables were generated based on the 1,000 bootstrap sequences using Dnadist. The bootstrap value was calculated using the Neighbors and Consens programs. For the maximum parsimony and maximum likelihood methods, Dnapars and Dnaml were used instead of Neighbors, respectively.

## Electronic supplementary material

Supplementary Info

Additional File 1

Additional File 2

Additional File 3
