# Supplementary material for: Serotyping dengue virus with isothermal amplification and a portable sequencer
Source: Sci Rep. 2017 Jun 14;7:3510. doi: 10.1038/s41598-017-03734-5 (PMC5471244; doi:10.1038/s41598-017-03734-5)
Supplement: Supplementary file 1 — Supplementary Info [file 41598_2017_3734_MOESM1_ESM.pdf]

Supplementary Files

Serotyping dengue virus with isothermal amplification and a portable sequencer

Junya Yamagishi, Lucky R. Runtuwene, Kyoko Hayashida, Arthur E. Mongan, Lan Anh Nguyen Thi, Linh Nguyen Thuy, Cam Nguyen Nhat, Kriengsak Limkittikul, Chukiat Sirivichayakul, Nuankanya Sathirapongsasuti, Martin Frith, Wojciech Makalowski, Yuki Eshita, Sumio Sugano, Yutaka Suzuki

a

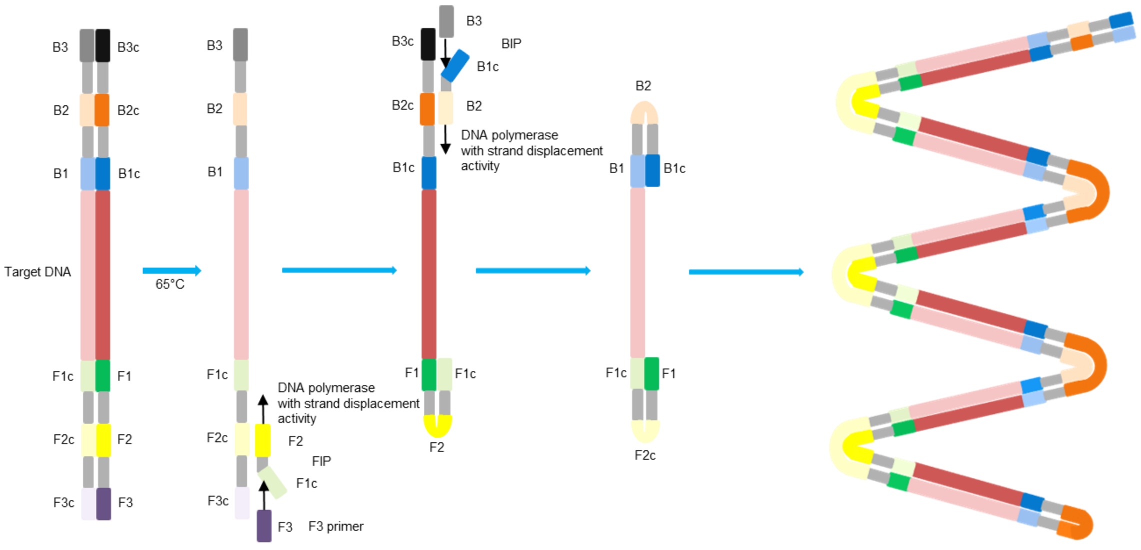

b

```
Den1 GAGTGATCCCGAAGGGGCACTCTGGTAAGCCAACTCATTCACAAAATAAAGGAAAATAAAAAATCAAACAAGGCAAGAAGTCAGGCCGGATTAAGCCATAGCACGGTAAGAG

Den2 AGAGGAAGAAGCAGGAGTTCTGTGGTAGA-------------------AAGCAAAACTAACATGAAACAAGGCTAGAAGTCAGGTCGGATTAAGCCATAGTACGGAAAAAA

Den3 AGAGGAGTCGGAGGGAGCCATTTGGTAAAC-----------------GTAGGAAGTGGAAAA------GAGGCTAACTGTCAGgccaccttaagccacagtaCGgaagaag
                                                                                         >-F3--------------->  |-FIP2-
Den4 TTCAGAGAGTGAAGGAGTTCTGTAATTACC--------------------------------------------------------------AACAACAA-ACACCAAAGG

         **    *  ** *   *  *  *                                                                   * * * *  **   *

Den1 CTATGCTGCCTGTGAGCCCCGTCCAAGGACGT---AAAATGAAGTCAGGCCG-----AAAGCCACGGTTCGAGCAAGCCGTGCTGCCTGTAGCTCCATC-GT----GGGGAT

Den2 CTATGCTACCTGTGAGCCCCGTCCAAGGACGTT---AAAAGAAGTCAGGCCATCATAAATGCCATAGCTTGAGTAAACTATGCAGCCTGTAGCTCCACCTGA----GAAGGT

Den3 ctgtgctgcctgTGagccccgtccaaggACGTTAAAAGAAgaagtcaggcccc----aaagccaCGGTTTGAGCAAACCGTGCTGCctgtagctccgtc-gt----ggggaC
     ----------->  <-FLP--------<            |-FIP1-----------------<                      >-BIP1------------------|
Den4 ctat----------------------------------tgaagtcaggccacT---TGTGccacggtttgagcaaaccGTGctgcctgtagctccgccAATaatgggaggc
     >-F3------------------------------------------------>        |-FIP2----------->   <-FLP----------->   |---FIP1--
     ** *                                    ***********         ****  * * *** ** *  *** ************  *       *  *

Den1 GTAAAAA-CCC--GGgaggctgcaaaccatggaaGctgtacgcatggggtagcAgactagtggttagaggagACCcctccc----aagacacaacgcagcAGC--GGGGccc
                    >-F3--------------> |-FIP2-----------> <-FLP------------<   |-FIP1-------------------<        >--
Den2 GTAAAAAATCC--GGGAGGCCACAAACCAtggaagctgtacgcatggCGtagtggactagcggttagaggAGAcccctccctt--acaaatcGcagcaacaat--gggggcc
                                  >-F3------------->  |-FIP2-------------->   <-FLP-------------< |-FIP1-------------
Den3 GTAAAA--CCT--GggaggctgcaaactgtgGAAGCTGTACgcacggtgtagcagactagCGGTTAGAGGAGACccctccc----atgacacaacGCAGCAGC--GGGGCCC
                   >-BLP----------->          <-BIP2------------|              <-B3----------------<
Den4 gtaataatccccaGGGAGGCCATGCGCCACGGAAGCTGTACGCGTGGCATATTGGACTAGCggttagaggagacccctcccAtcactgataaaacgcagCAAAAGGGGGCCc
     ------------<                                                >-BIP1-------------| >-BLP----------->            <
     **** *   *   *******      *   *************  **  **   ****** *******************        *      *** **    **** **

Den1 aa-caccaggggaagctgtACCCTggtggtaaggactagaggTTAGAGGAGACcccccgcacaacaacaaaCAGCATATTGACGCTGGGagagaccagagatcctgctgTCT
     BIP1------------|       >-BLP------------>           <-BIP2-----------|                  <-B3---------------<
Den2 caaGGCGAGATGAAGCTGTAGTCTCGCTGGAAGGACTAGAggttagaggagacccccccga-aACAAAAAACAgcatattgacgctgggaAAGAccagagatcctgctgtct
     --<                                     >-BIP1----------------|          >-BLP----------->    <-BIP2------------
Den3 GAGCACTGAGGGAAGCTGTACCTCCTTGCAAAGGACTAGAGGTTAGAGGAGACCCCCCGCA-AATAA-AAACAGCATATTGACGCTGGGAGAGACCAGAGATCCTGCTGTCT

Den4 gaagccaggaggaagctGTACTCCTggtggaaggactagaggtTAGAGGAGACCCCCCCAA-CACAA-AAACAGCATATTGACGCTGGGAAAGACCAGAGATCCTGCTGTCT
     -BIP2-----------|        <-B3-------------<
      *   *     *********          ****************************  *  * ** ********************** *********************

Den1 CTACAGCATCATTCCAGGCACAGAACGCCAAAAAATGGAATGGTGCTGTTGAATCAACAGGTTCT

Den2 ccTcagcatcattccaggcacAGAACGCCAGAAAATGGAATGGTGCTGTTGAATCAACAGGTTCT
     -| <-B3-------------<
Den3 CCTCAGCATCATTCCAGGCACAGAACGCCAGAAAATGGAATGGTGCTGTTGAATCAACAGGTTCT

Den4 CTGCAACATCAATCCAGGCACAGAGCGCCGCAAGATGGATTGGTGTTGTTGATCCAACAGGTTCT

     *  ** ***** ************ ****  ** ***** ***** ******  ***********
```

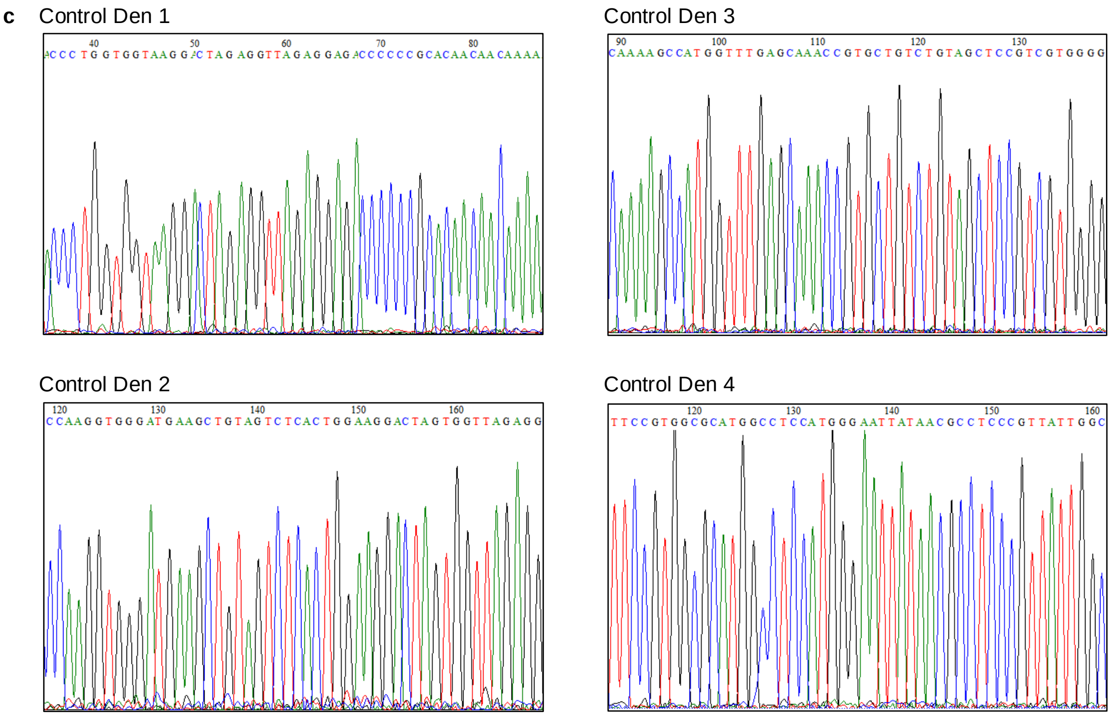

Figure S1 | The schematics of LAMP.
a, Schematic representation of LAMP. Using six pair of primers, LAMP amplify the target DNA or RNA into a ladder configuration. Briefly, the primers form the indicated hairpin structure, which allows turn back priming of the DNA polymerase in an isothermal reaction. For further details, see the reference. b, Primers used for the LAMP amplification for each serotype. Asterisks at the bottom indicate the common bases between the serotypes. For the alignment, the genome sequences of D1-D4 are aligned using ClustalW. **c,** Sanger sequencing of the control viral RNAs.

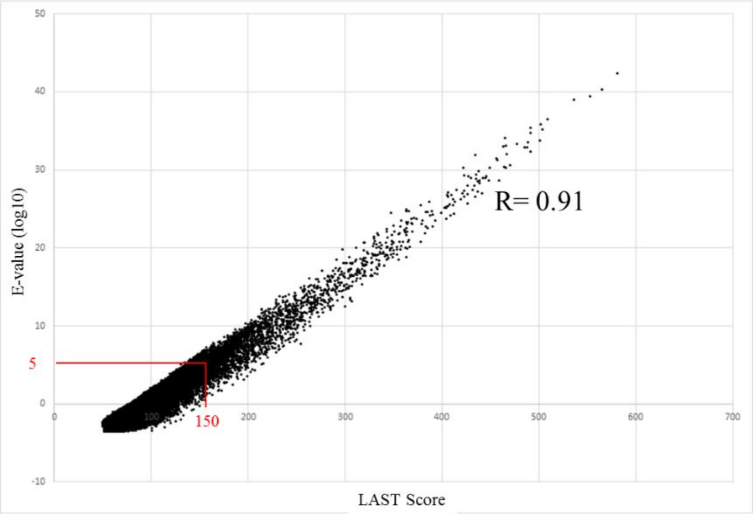

Figure S2 | The correlation between LAST score and e-value.
Each sequence obtained from a sequence run had an e-value calculated using the LAST option and this value was compared with its LAST score. The e-value corresponding to a LAST score of 150 is highlighted. Pearson's correlation is also shown.

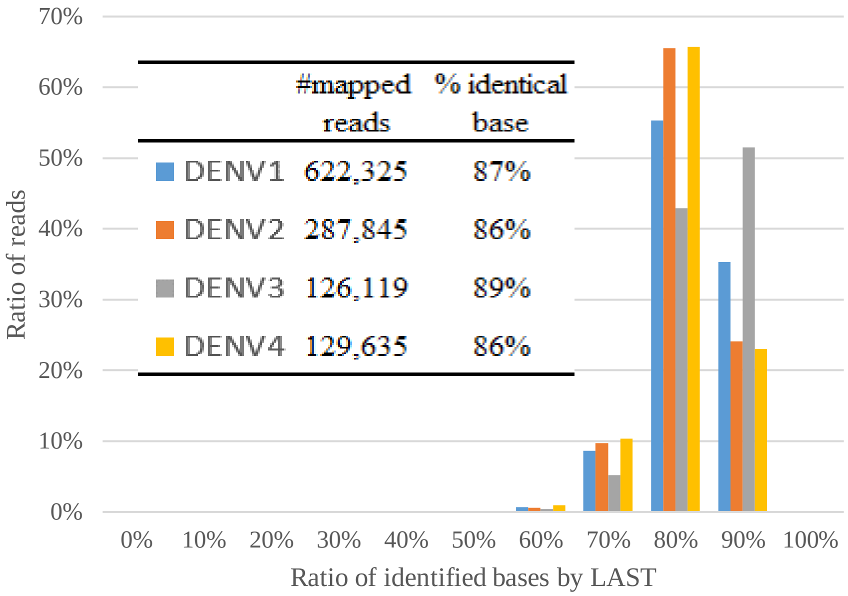

Figure S3 | Sequencing result of control samples obtained by newer version flow cell.
Accuracy of the sequencing obtained by flow cell version 9.4 was calculated by considering the matched base between each sequence and the corresponding reference genome.

a

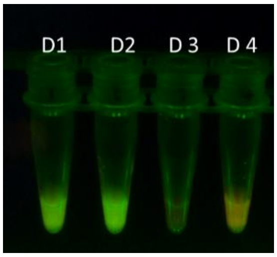

b

```
Query: B24
TargetSubject: Den1
sstart-send: 10489-10550
MinScore: 450
Subject:CTGT-ACGCATGGGGTAGCA-GACTAGTGGTTAGAG--GAGACCCCTCCCAAGACACAACGCAGCA
read1  :****-**********CCC*--***************--*****G**********************
read2  :****G***************-***************--*******-********************
read3  :****-***************A***************--*-**T***CT***CA*************
read4  :  **-***************-A--************--****T*******************CCG*
read5  :****-******-********-***************AG*******--T******************
read6  :****-*****CTC*******-***************--*******-***AC***************

Query: B54
TargetSubject: Den3
sstart-send: 10321-10382
MinScore: 750
Subject:GAAGAAGCTGTGCTGC-CTGTGAGCCC-CGTCCA--AGGACGTTAAAAGAAGAAGTCAGGCCCCAA
read1  :*************A**-**********-******CATA*************************A**
read2  :****G********A**-**********--*****--***************************A*C
read3  :*************A**C**********-******--***************************A**
read4  :*************A**-*******-**-******--***************************A**
read5  :*************A**-**********C******--****************---********A**
read6  :*************A**-****-****---*****--***************************A**
read7  :*************A**-**********-******--***************************A**
read8  :*************A**-**********-******--***G***********************-**

Query: B49
TargetSubject: Den2
sstart-send: 10489-10550
MinScore: 670
Subject:TAGTGGACT-AGC-GGTT-AGAGGAGACCCCTCCCTTACAAATCGCAGCAACAAT-GGGGGCCCAA
read1  :*********-***-****T***********T--*******G***A**********-**********
read2  :*********-***-****--********************G***A**********-**********
read3  :****-****-***-****-*********************G***A**TA******-**********
read4  :*********-***G****-*********************G***A**********-**********
read5  :           **-****-***************A*****G***A**********-**********
read6  :     ****-***-****-T********************G***A**********-**********
read7  :*********-***-*-**-*****C***************G**************-**********
read8  :  *******G***-*T*G-**G******************G***A**********-**********
read9  :  *******-***-****-*********************G***A**********C**********
read10 :*********-***-*-**-**************G*G**-*G***A**********-**********
read11 :  *******-***-****-***********-*********G***A********-*-**********

Query: B17
TargetSubject: Den4
sstart-send: 10312-10369
MinScore: 730
Subject:CCACGGTTTGAGCA-AACCGTGCTGCCTGTAGCTCCGCCAATAATGGGAGG-----CGT-AATAA
read1  :******C*******-************************************-----***-T****
read2  :******C*******-************************************-----***-T****
read3  :******C*******-************************************-----***-T****
read4  :******C*******-************************************GCGGG***-TGACC
read5  :******C*******-*********************A*-************-----***T*CCCT
read6  :******C*******-**G*****************************-***-----***-T****
read7  :******C*******-***********-*********C**************-----***-T*CGT
read8  :******C******CC**A*********************************-----***-T****
```

Figure S4| MinION sequencing of clinical samples.
a, Examples of erroneous LAMP results. Note that LAMP results by their own were not always conclusive. **b,** Example of clinical samples sequencing. The highest score reads are shown here. These reads have approximately 95% in fidelity.

a

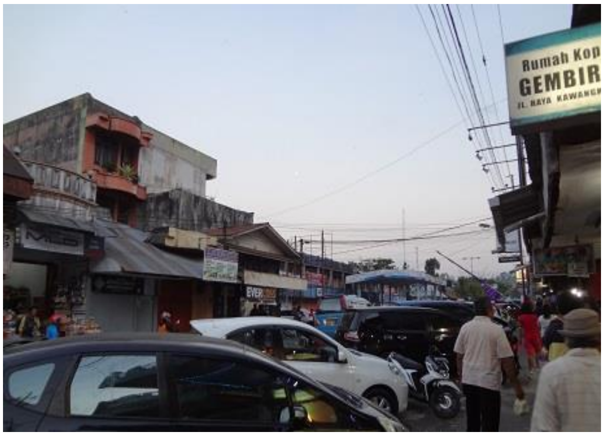

b

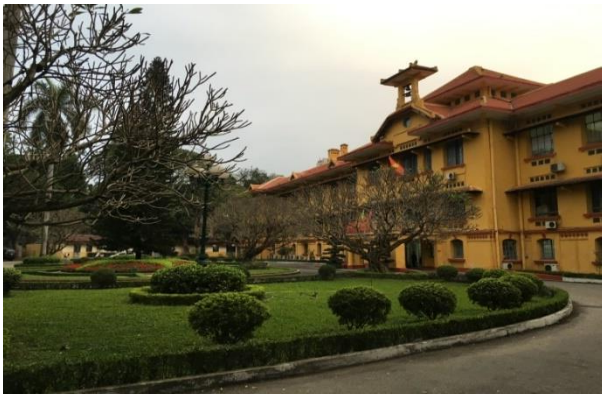

c

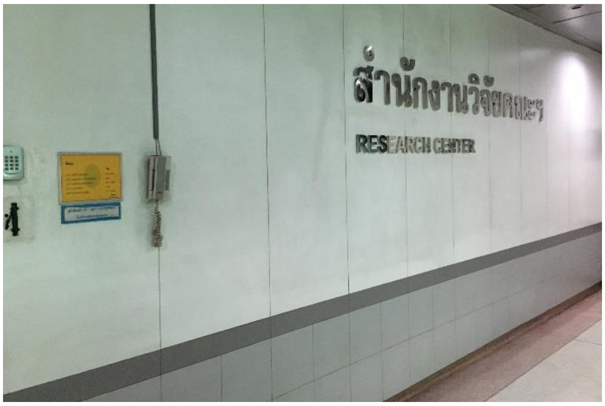

Figure S5 | On-site MinION sequencing
The photos of the outlook of the clinics in Indonesia (**a**), Vietnam (**b**), and Thailand (**c**).

a

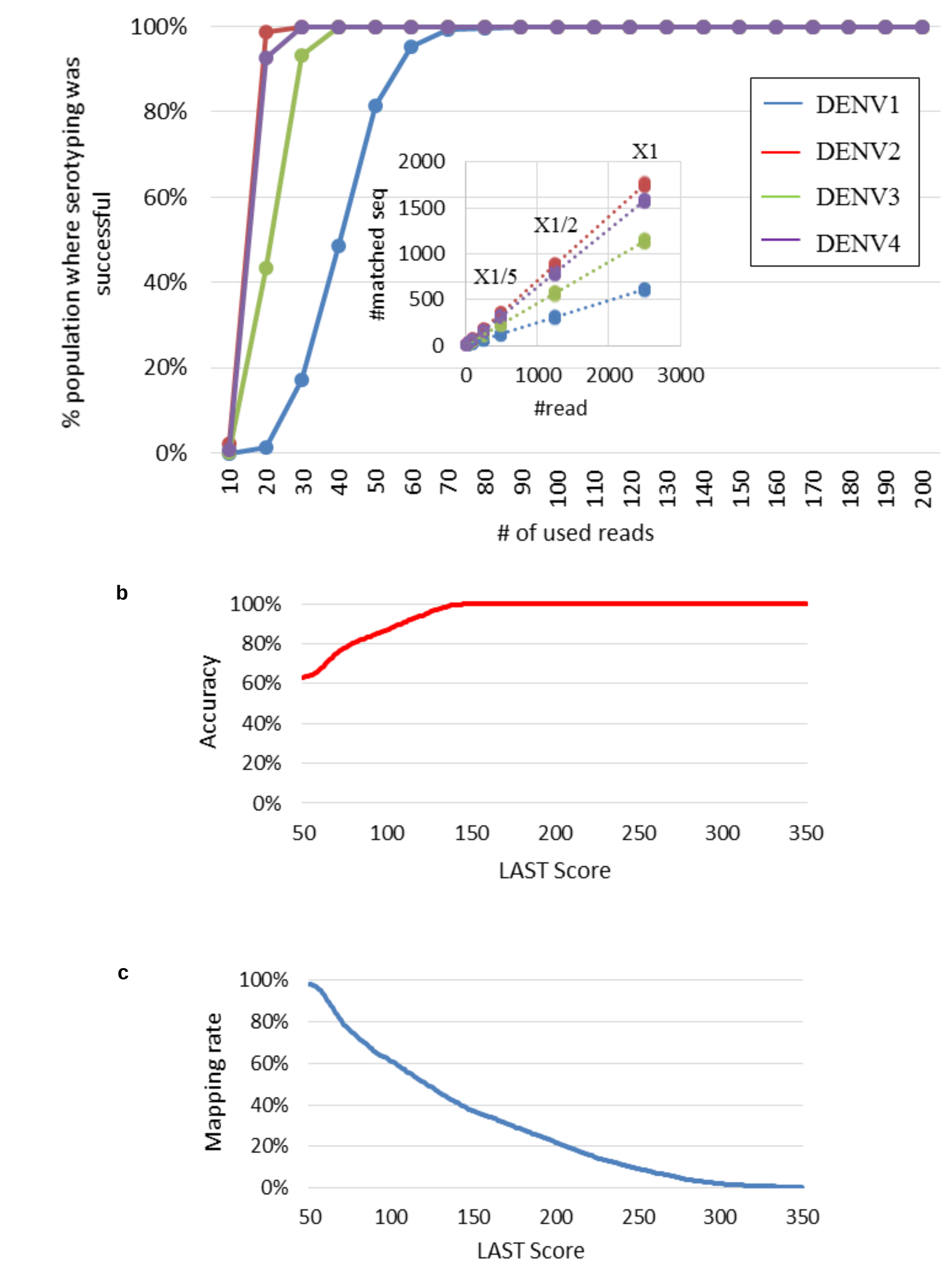

Figure S6 | Scaling the analysis with barcode oligos.
a, Results from the simulation analysis regarding the number of sequence reads necessary to include more than ten sequence reads supporting the serotyping of the indicated serotype. **b, c**, The precision (**b**) and recall (**c**) that separate barcoding oligos.

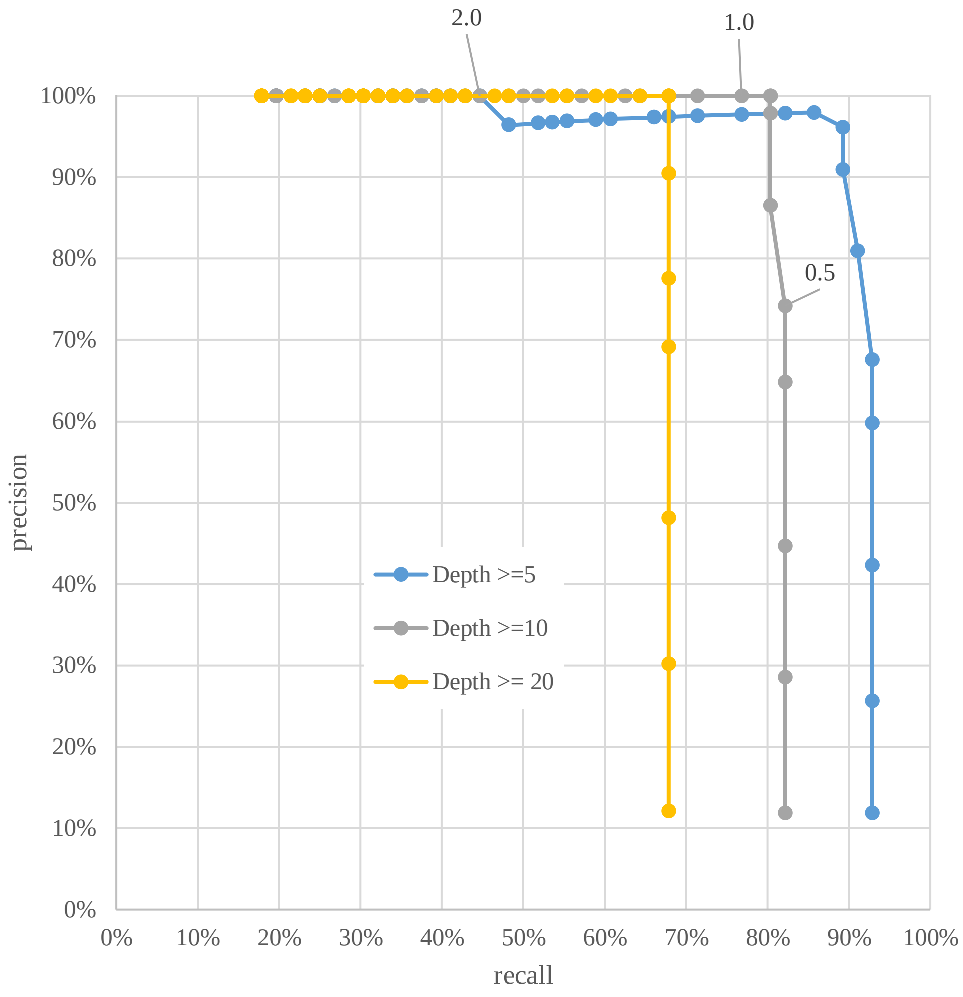

Figure S7 | Precision plotted against recall
We employed threshold 2.0 for our analysis, which is conservative with a recall rate lower than 50%. Threshold 1.0, on the other hand, gives varied results among different datasets. For that reason, we did not change our threshold for subsequent analysis.

a

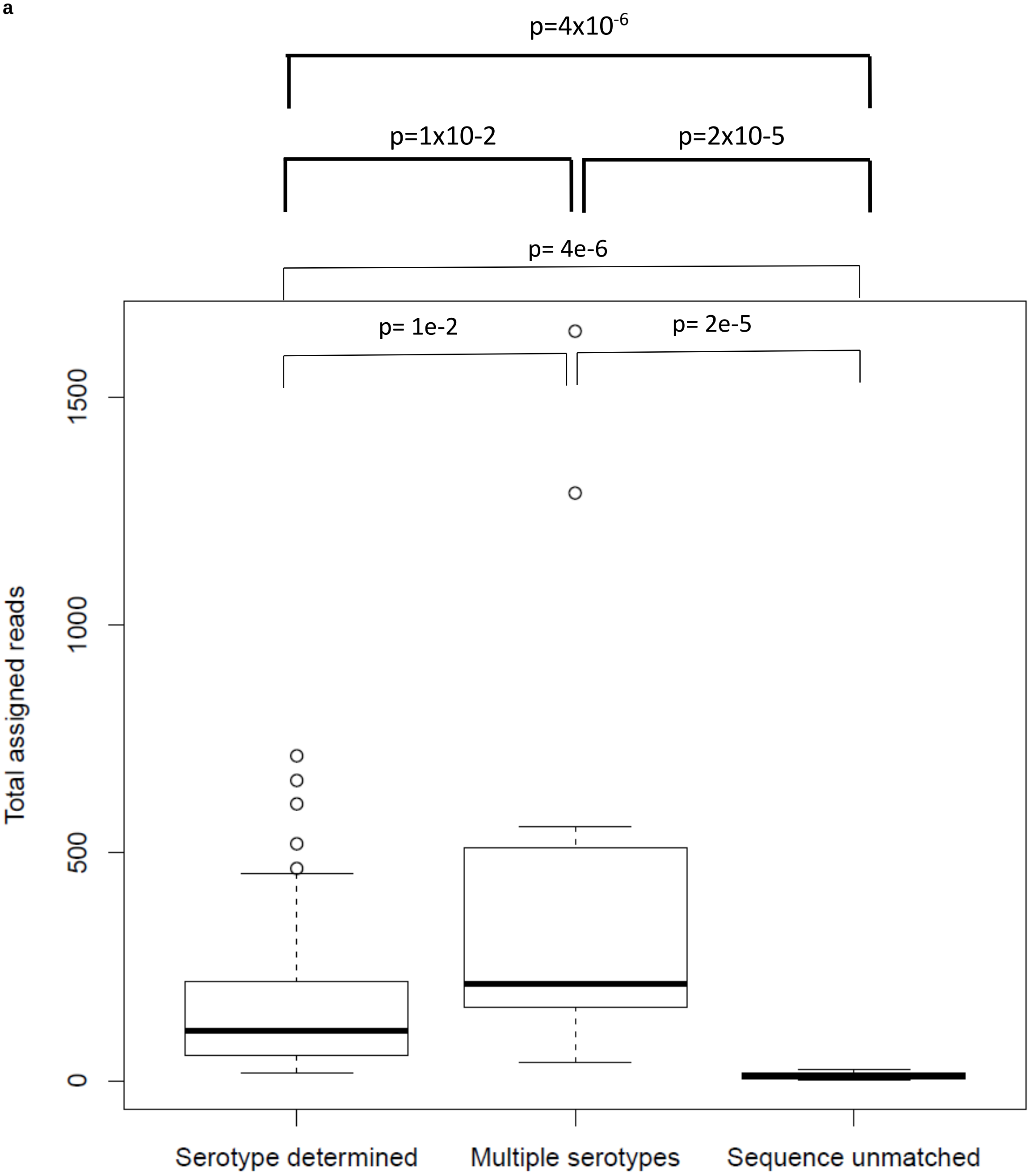

b

template	D1:D4	1:0	1:1	1:1/10	1:1/100	1:1/1,000	1:1/10,000	
primer	D1	1,825	2,550	2,575	2,250	3,350	2,850	(ng)
primer	D4	25	4,825	2,800	25	25	0	

c

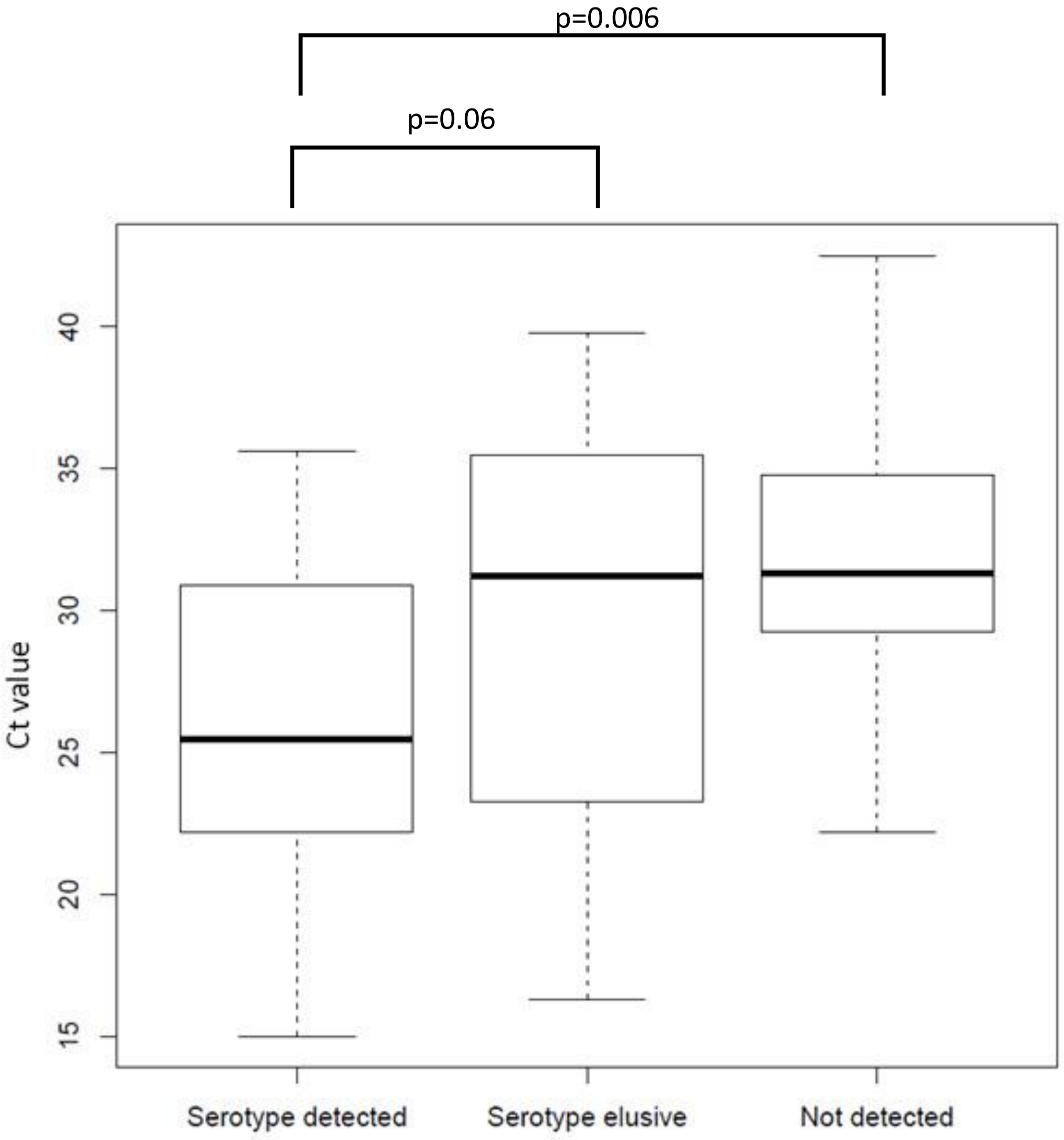

p=0.006
p=0.06
Ct value
15
20
25
30
35
40
Serotype detected
Serotype elusive
Not detected

d

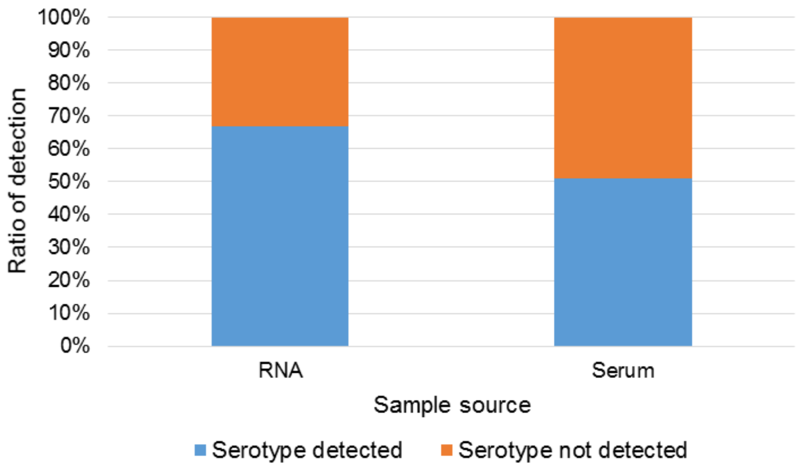

100%
90%
80%
70%
60%
50%
40%
30%
20%
10%
0%
Ratio of detection
RNA
Serum
Sample source
Serotype detected
Serotype not detected

e

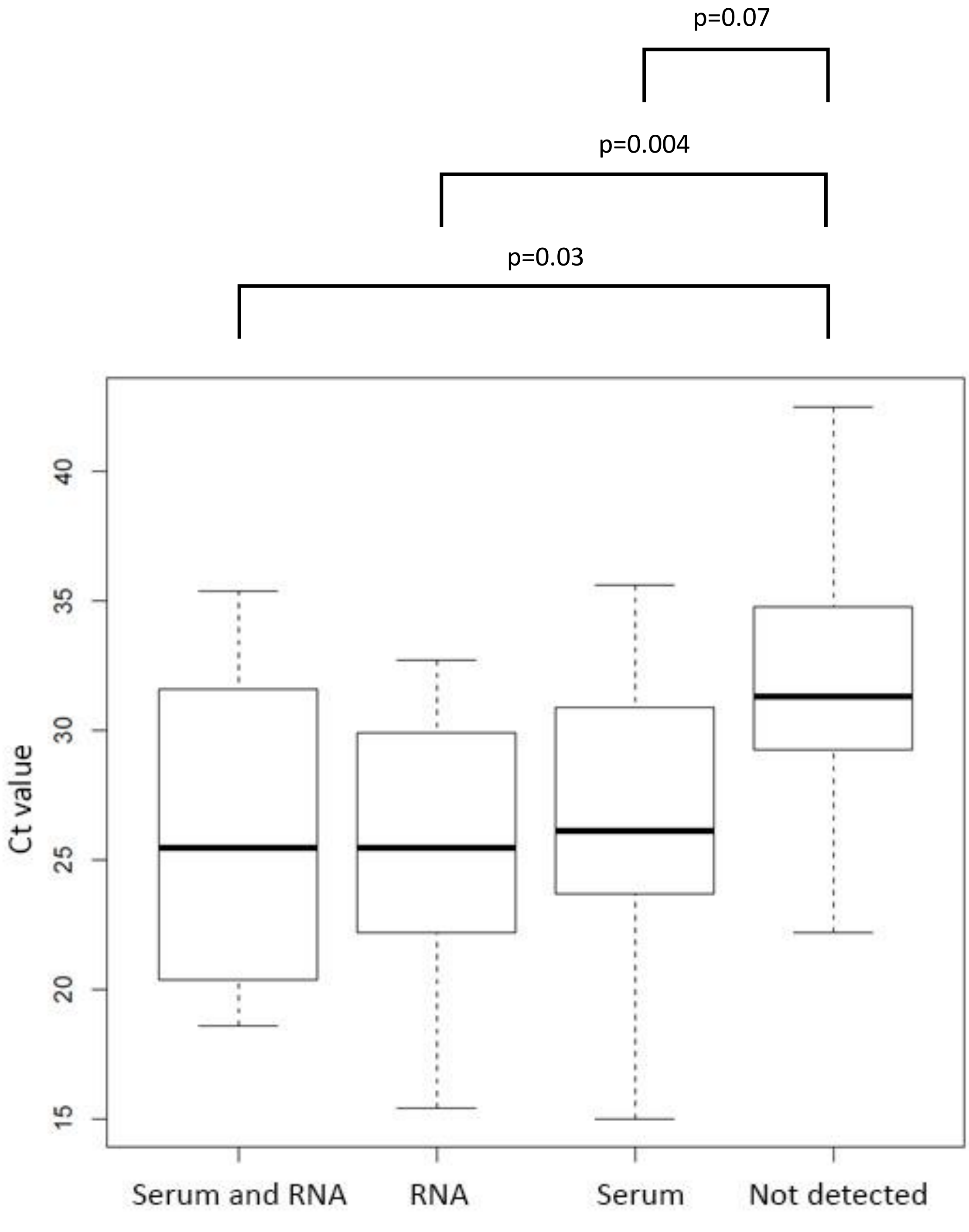

p=0.07
p=0.004
p=0.03
Ct value
40
35
30
25
20
15
Serum and RNA
RNA
Serum
Not detected

f

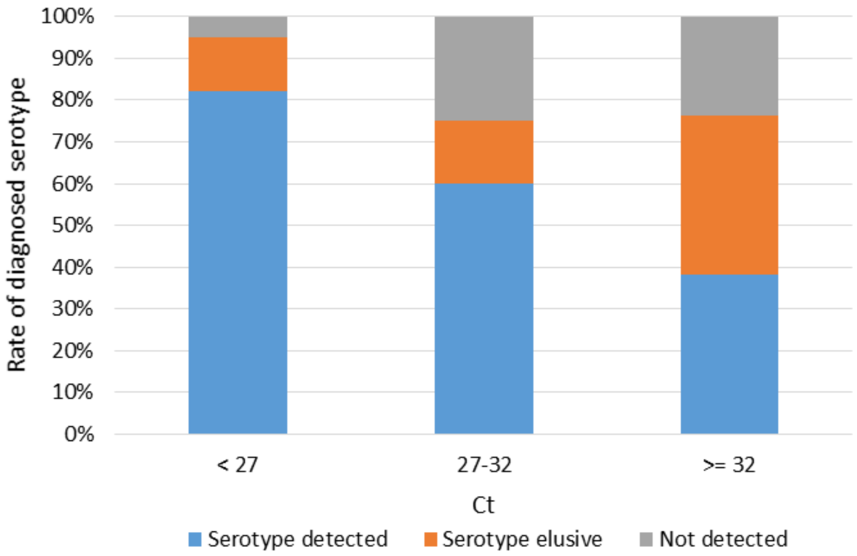

Figure S8 | Possible causes of the ambiguous detections.

a, The number of sequence reads for the indicated population. The difference in statistical significance was evaluated by the Wilcoxson's singed rank test and shown on the plot. **b,** Non-specific amplification of the contaminated templates. Amounts of the LAMP amplicons using the indicated templates and the primers are shown. Templates were serially mixed with the indicated ratios between the D1 and D4 viral RNA with the total amount of 400 p.f.u.. Note that remnant amount of amplicons of D4 was observed even at the 1/100 dilutions or less. **c,** The detection rate is correlated with viral titer, which is shown here with Ct value from qRT-PCR of DENV E protein. **d,** Detection rate is slightly correlated with sample purity, with purified virus RNA shows slightly better detection rate. **e, f,** Detection rate is correlated with viral titer (in this case shown by Ct value), with higher viral titer (lower Ct value) gives a better detection rate.

a

Sample ID	Sequencing mode	Category	Diagnosed serotype	Total reads	Total mapped reads	Total reads mapped to the corresponding serotype	Mapped ratio	Average sequence identity
Control D1	2D	Control	D1	4,060	2,164	2,155	100%	77%
Control D2	2D	Control	D2	9,260	6,719	6,617	98%	79%
Control D3	2D	Control	D3	9,750	7,885	7,873	100%	82%
Control D4	2D	Control	D4	2,758	2,014	2,010	100%	78%
Control D1	Template	Control	D1	22,510	1,160	1,133	98%	70%
Control D2	Template	Control	D2	24,079	3,623	3,375	93%	69%
Control D3	Template	Control	D3	22,842	6,557	6,499	99%	71%
Control D4	Template	Control	D4	5,579	967	967	100%	70%
Control D1	Complement	Control	D1	5,943	41	41	100%	68%
Control D2	Complement	Control	D2	11,450	577	527	91%	69%
Control D3	Complement	Control	D3	12,436	755	751	99%	69%
Control D4	Complement	Control	D4	3,242	62	62	100%	70%

b.

Program	Sample ID	Sequencing mode	# of total reads	# of mapped reads	# of reads mapped to the corresponding serotype	Mapped ratio
LAST	Control D1	2D	4,060	2,164	2,155	100%
	Control D2	2D	9,260	6,719	6,617	98%
	Control D3	2D	9,750	7,885	7,873	100%
	Control D4	2D	2,758	2,014	2,010	100%
	Control D1	Template	22,510	1,160	1,133	98%
	Control D2	Template	24,079	3,623	3,375	93%
	Control D3	Template	22,842	6,557	6,499	99%
	Control D4	Template	5,579	967	967	100%
	Control D1	Complement	5,943	41	41	100%
	Control D2	Complement	11,450	577	527	91%
	Control D3	Complement	12,436	755	751	99%
	Control D4	Complement	3,242	62	62	100%
GraphMap	Control D1	2D	4,060	3,073	2,606	85%
	Control D2	2D	9,260	7,686	5,293	69%
	Control D3	2D	9,750	8,218	6,911	84%
	Control D4	2D	2,758	2,186	1,796	82%
	Control D1	Template	22,510	12,949	9,561	74%
	Control D2	Template	24,079	13,763	7,880	57%
	Control D3	Template	22,842	15,747	11,867	75%
	Control D4	Template	5,579	2,985	2,116	71%
	Control D1	Complement	5,943	1,908	1,052	55%
	Control D2	Complement	11,450	5,439	2,706	50%
	Control D3	Complement	12,436	5,987	3,566	60%
	Control D4	Complement	3,242	1,141	623	55%
SSEARCH	Control D1	2D	4,060	2,136	2,115	99%
	Control D2	2D	9,260	6,658	6,511	98%
	Control D3	2D	9,750	7,833	7,800	100%
	Control D4	2D	2,758	2,047	2,044	100%
	Control D1	Template	22,510	3,272	3,217	98%
	Control D2	Template	24,079	6,899	6,566	95%
	Control D3	Template	22,842	11,050	10,921	99%
	Control D4	Template	5,579	1,992	1,992	100%
	Control D1	Complement	5,943	189	187	99%
	Control D2	Complement	11,450	1,547	1,489	96%
	Control D3	Complement	12,436	1,963	1,940	99%
	Control D4	Complement	3,242	278	278	100%

c.

Control, D1	4,060				
Cut-off (Score)	#Seq (D1)	#Seq (D2)	#Seq (D3)	#Seq (D4)	%Seq (D1)
200	1,502	0	2	0	100%
150	2,155	0	6	3	100%
50	3,245	207	298	207	82%

Clinical, D1	3,156	2015_B24			
Cut-off (Score)	#Seq (D1)	#Seq (D2)	#Seq (D3)	#Seq (D4)	%Seq (D1)
200	407	0	1	0	100%
150	1,087	0	4	0	100%
50	2,607	138	207	125	85%

Control, D2	9,260				
Cut-off (Score)	#Seq (D1)	#Seq (D2)	#Seq (D3)	#Seq (D4)	%Seq (D2)
200	1	5,898	6	39	99%
150	11	6,617	12	79	98%
50	413	7,556	419	713	83%

Clinical, D2	4,680	2015_B49			
Cut-off (Score)	#Seq (D1)	#Seq (D2)	#Seq (D3)	#Seq (D4)	%Seq (D2)
200	2	3,088	3	25	99%
150	9	3,534	4	63	98%
50	185	3,931	134	378	85%

Control, D3	9,750				
Cut-off (Score)	#Seq (D1)	#Seq (D2)	#Seq (D3)	#Seq (D4)	%Seq (D3)
200	4	0	6,965	0	100%
150	11	1	7,873	0	100%
50	251	256	8,929	221	92%

Clinical, D3	5,598	2015_B54			
Cut-off (Score)	#Seq (D1)	#Seq (D2)	#Seq (D3)	#Seq (D4)	%Seq (D3)
200	0	0	2,168	0	100%
150	11	0	2,666	0	100%
50	512	537	3,960	421	73%

Control, D4	2,758				
Cut-off (Score)	#Seq (D1)	#Seq (D2)	#Seq (D3)	#Seq (D4)	%Seq (D4)
200	0	0	0	1,643	100%
150	0	0	4	2,010	100%
50	90	68	108	2,464	90%

Clinical, D4	4,220	2015_B17			
Cut-off (Score)	#Seq (D1)	#Seq (D2)	#Seq (D3)	#Seq (D4)	%Seq (D4)
200	0	0	0	2,341	100%
150	0	1	3	2,842	100%
50	208	175	228	3,533	85%

Table S1 | Statistics of the “2D”, “template” and “complement”.
a, The statistics of MinION sequencing showed 5,000-20,000 reads were obtained for “template” and “complement” and 2,500-10,000 reads for “2D”. Approximately 91-100% reads were mapped to the respective serotype reference. **b** The statistics of analysis with GraphMap and SSEARCH compared to LAST. **c,** The statistics of various LAST score parameters with control and clinical samples mapped to their respective serotype.

Sample ID	Primer set	Den1	Den2	Den3	Den4
Control D1	D1		144	613	150
Control D2	D2	275		245	1034
Control D3	D3	1880	756		2925
Control D4	D4	24	44	29	
B24	D1		31	135	23
B49	D2	205		168	428
B54	D3	478	51		966
B17	D4	46	98	66	
B25	D1		67	251	55
B03	D1		34	158	27
11	D1		56	187	39
01	D1		4	35	5
21	D1		3	49	1
17	D1		23	136	18
B28	D3	213	48		411

Table S2 | The results from serotyping that exclude the primer sites.
The number of sequences that showed base matches to the diagnosed serotype for all of the positions that are different between the diagnosed serotypes and indicated serotypes. Note that no sequences matched at all the positions of the candidate serotypes when compared to the diagnosed serotypes.

a.

Sample ID	Sequencing mode	Category	Diagnosed serotype	Total reads	Total mapped reads	Total reads mapped to the corresponding serotype	Mapped ratio	Average sequence identity
Control D1	2D	Control	D1	650,232	622,563	622,325	100%	87%
Control D2	2D	Control	D2	296,751	288,743	287,845	99%	86%
Control D3	2D	Control	D3	129,955	126,193	126,119	100%	89%
Control D4	2D	Control	D4	133,968	129,643	129,635	100%	86%
Control D1	Template	Control	D1	973,069	868,796	867,602	100%	82%
Control D2	Template	Control	D2	498,409	441,176	437450	99%	80%
Control D3	Template	Control	D3	200,498	178,831	179,391	100%	84%
Control D4	Template	Control	D4	223,864	184,034	184,008	100%	80%
Control D1	Complement	Control	D1	703,513	587,770	586,571	100%	80%
Control D2	Complement	Control	D2	331,234	281,245	276,948	98%	78%
Control D3	Complement	Control	D3	144,910	124,163	123,572	100%	82%
Control D4	Complement	Control	D4	160,309	126,841	126,825	100%	79%

b.

Sample ID	Sequencing mode	Total reads	Total mapped reads (score=150)	Total reads mapped to the corresponding serotype (score=150, mismap=1)				Ratio
				DENV1	DENV2	DENV3	DENV4	
Control DENV1	2D	650,232	622,563	622,325	5	37	196	100%
Control DENV2	2D	296,751	288,743	20	287,845	140	738	100%
Control DENV3	2D	129,955	126,193	67	4	126,119	3	100%
Control DENV4	2D	133,968	129,643	0	2	6	129,635	100%

c.

Sample ID	Sequencing mode	Category	Diagnosed serotype	Total reads	Number of mapped reads	Number of reads mapped to the corresponding serotype	Percent ratio	Average sequence identity
B24	2D	Clinical representative	DENV1	20,854	18,025	18,022*	100%	71%*
B49	2D	Clinical representative	DENV2	274,669	241,189	237,421	98%	81%
B54	2D	Clinical representative	DENV3	283,144	264,987	264,666	100%	88%
B17	2D	Clinical representative	DENV4	263,889	230,336	230,317	100%	80%

Table S3 | Sequencing result of control samples obtained by newer version flow cell.
a, Newer version of flow cell shows higher total reads yield and sequence identity when sequencing control samples. **b,** Almost all of the "2D' reads were successfully mapped to the respective serotype. **c,** Sequence of representative clinical samples also shows better statistics to almost all samples. One sample (shown by asterisk) has worse sequence identity compared to the former version. This is due to unsuccessful LAMP amplification.

a

Category	Sample	Total aligned match	Match	Insertion	Deletion	Mismatch	% match	% insertion	% deletion	% mismatch
Control	D1	236,384	181,778	4,324	21,899	28,383	77%	2%	9%	12%
Control	D2	916,128	720,072	24,809	57,943	113,304	79%	3%	6%	12%
Control	D3	981,318	801,880	33,231	45,415	100,792	82%	3%	5%	10%
Control	D4	258,734	202,995	8,876	12,347	34,516	78%	3%	5%	13%
Total		2,392,564	1,906,725	71,240	137,604	276,995	80%	3%	6%	12%

b

Reference genome

		A	C	G	T
MinION reads	A		28,958	31,650	11,003
	C	21,260		25,959	17,772
	G	29,528	25,354		10,816
	T	15,730	33,672	25,293	
	-	36,082	45,765	41,204	14,553

Table S4 | Patterns of possible sequence errors in the sequence reads using the control samples.
a, The overall frequency of the mismatch pattern**. b,** The detailed base substitution patterns.

a

Sample name	Age	Sex	Clinical Evidence	RT-PCR	MinION
Healthy_01	40	F	NA	ND	ND
Healthy_02	66	M	NA	ND	ND
Healthy_03	66	F	NA	ND	ND
Healthy_04	70	F	NA	ND	ND
Healthy_05	33	F	NA	ND	ND
Healthy_06	77	M	NA	ND	ND
Healthy_08	20	M	NA	ND	ND
Healthy_09	66	M	NA	ND	ND
Healthy_10	17	M	NA	ND	ND
Healthy_11	10	F	NA	ND	ND
Healthy_12	66	F	NA	ND	ND
Healthy_13	10	M	NA	ND	ND
Healthy_14	37	M	NA	ND	ND
Healthy_15	37	F	NA	ND	ND
Healthy_16	67	M	NA	ND	ND
Healthy_17	75	M	NA	ND	ND
Healthy_18	25	M	NA	ND	ND
Healthy_19	30	M	NA	ND	ND
Healthy_20	59	M	NA	ND	ND
Kashiwa_01	45	M	NA	ND	ND
Kashiwa_02	42	F	NA	ND	ND
Kashiwa_03	26	F	NA	ND	ND
Kashiwa_04	33	F	NA	ND	ND
Kashiwa_05	33	F	NA	ND	ND
Kashiwa_06	33	F	NA	ND	ND
Kashiwa_07	42	F	NA	ND	ND
Kashiwa_08	38	F	NA	ND	ND
Kashiwa_09	34	M	NA	ND	ND
Kashiwa_10	58	F	NA	ND	ND
Kashiwa_11	23	M	NA	ND	ND
Kashiwa_12	48	F	NA	ND	ND
Kashiwa_13	41	M	NA	ND	ND
Kashiwa_14	29	F	NA	ND	ND

b

Sample ID	RT-PCR	MinION
B24	D1	D1
B49	D2	D2
B54	D3	D3
B17	D4	D4
01	ND	D1
11	ND	D1
17	NA	D1
21	ND	D1
B03	D1	D1
B25	D1	D1
B28	D3	D3

c

Sample origin	Total samples	RT-PCR		MinION (same sample as RT-PCR)		
		Detected	Not detected	Detected	Not detected	Serotype consistent with RT-PCR
Indonesia	11	7	4	11	0	7
Thai	12	12	0	12	0	12
Vietnam	26	24	2	26	0	24

d

Sample ID	Diagnosed serotype	Total aligned reads	Match	Insertion	Deletion	Mismatch	% match	% ins	% del	% mismatch
B24	D1	101,438	75,453	7,708	6,984	11,293	74%	8%	7%	11%
B49	D2	477,863	371,774	12,780	29,674	63,635	78%	3%	6%	13%
B54	D3	336,605	269,114	11,940	18,798	36,753	80%	4%	6%	11%
B17	D4	419,953	319,287	16,861	22,274	61,531	76%	4%	5%	15%
1	D1	14,465	11,080	328	1,302	1,755	77%	2%	9%	12%
11	D1	60,949	47,116	1,204	5,704	6,925	77%	2%	9%	11%
17	D1	43,352	33,500	979	3,666	5,207	77%	2%	8%	12%
21	D1	18,774	14,337	514	1,647	2,276	76%	3%	9%	12%
B03	D1	40,603	31,338	1,210	2,999	5,056	77%	3%	7%	12%
B25	D1	63,718	50,075	1,570	4,529	7,544	79%	2%	7%	12%
B28	D3	114,623	92,477	4,374	5,576	12,196	81%	4%	5%	11%
Total		1,692,343	1,315,551	59,468	103,153	214,171	78%	4%	6%	13%

e

Sample	Serotype	SNP called by MinION	SNP called by Illumina
B24	D1	Den1:10553:G:A	Den1:10581:C:G
B49	D2	Den2:10553:C:T<br>Den2:10555:A:G<br>Den2:10560:A:G<br>Den2:10573:G:A	Den2:10555:A:G<br>Den2:10560:A:G<br>Den2:10573:G:A
B54	D3	Den3:10391:T:C	Den3:10391:T:C
B17	D4	Not detected	Den4:10403:C:T<br>Den4:10415:T:C
01	D1	Not detected	Den1:10581:C:G
11	D1	Not detected	Den1:10581:C:G
17	D1	Not detected	Not detected
21	D1	Not detected	Not detected
B03	D1	Den1:10581:C:G	Not analyzed
B25	D1	Den1:10581:C:G	Den1:10581:C:G
B28	D3	Den3:10391:T:C	Den3:10391:T:C

f

Sample ID	Sequencing mode	Category	Diagnosed serotype	# total reads	# mapped reads	# of read mapped to the corresponding serotype	Mapped ratio	Average sequence identity
021	2D	Clinical Indonesian	D4	26	11	11	100%	72%
021	Template	Clinical Indonesian	D4	372	8	8	100%	67%
021	Complement	Clinical Indonesian	D4	66	0	0	-	-

g

	Illumina					RT-PCR						
	Matched	%	Unmatched	%	Total	Matched	%	Unmatched	%	Not detected	%	Total
Serotype determined	43	96%	2	4%	45	10	37%	1	4%	16	59%	27
Multiple serotype	6	86%	1	14%	7	0	0%	2	33%	4	67%	6
Serotype undetermined	NA		NA		NA	0	0%	0	0%	2	100%	2
Not detected	NA		NA		NA	0	0%	0	0%	8	100%	8
Total	49	94%	3	6%	52	10	23%	3	7%	30	70%	43

Table S5 | Statistics of the MinION sequencing for clinical samples.

a, Results of the analysis using healthy samples, from Indonesia (labeled as Healthy) and from Japan (labeled as Kashiwa). **b, c,** RT-PCR validation of the MinION results shows that MinION can detect dengue serotype when RT-PCR fails to detect the serotype. **d,** Patterns of the putative sequence errors are shown. **e,** The positions and patterns of the SNVs and their validations for the non-multiplexed samples. **f,** Data from one of the field-analyzed samples are similar with those of laboratory-analyzed. **g,** Illumina and RT-PCR validation of MinION results for the multiplexed samples. NA: Not analyzed; ND: Not detected.

a

for type 1	FIP	GCTGCGTTGTGTCTTGGGAGGTTTTCTGTACGCATGGGGTAGC
	BIP	CCCAACACCAGGGGAAGCTGTTTTTTTGTTGTTGTGCGGGGG
	FLP	CTCCTCTAACCACTAGTC
	BLP	GGTGGTAAGGACTAGAGG
	F3	GAGGCTGCAAACCATGGAA
	B3	CAGCAGGATCTCTGGTCTCT
for type 2	FIP	TTGGGCCCCCATTGTTGCTGTTTTAGTGGACTAGCGGTTAGAGG
	BIP	GGTTAGAGGAGACCCCCCCAATTTTGGAGACAGCAGGATCTCTGG
	FLP	GATCTGTAAGGGAGGGG
	BLP	GCATATTGACGCTGGGA
	F3	TGGAAGCTGTACGCATGG
	B3	GTGCCTGGAATGATGCTG
for type 3	FIP	TGGCTTTTGGGCCTGACTTCTTTTTTGAAGAAGCTGTGCAGCCTG
	BIP	CTGTAGCTCCGTCGTGGGGATTTTCTAGTCTGCTACACCGTGC
	FLP	CCTTGGACGGGGCT
	BLP	GGAGGCTGCAAACCGTG
	F3	GCCACCTTAAGCCACAGTA
	B3	GTTGTGTCATGGGAGGG
for type 4	FIP	TGGGAATTATAACGCCTCCCGTTTTTTCCACGGCTTGAGCAAACC
	BIP	GGTTAGAGGAGACCCCTCCCTTTTAGCTTCCTCCTGGCTTCG
	FLP	GGCGGAGCTACAGGCAG
	BLP	TCACCAACAAAACGCAG
	F3	CTATTGAAGTCAGGCCAC
	B3	ACCTCTAGTCCTTCCACC

b

for type 1	D1_FIP_ind1	GCTGCGTTGTGTCTTGGGAGGAAGAAAGTTGTCGGTGTCTTTGTGCTGTACGCATGGGGTAGC
	D1_FIP_ind2	GCTGCGTTGTGTCTTGGGAGGTCGATTCCGTTTGTAGTCGTCTGTCTGTACGCATGGGGTAGC
	D1_FIP_ind3	GCTGCGTTGTGTCTTGGGAGGGAGTCTTGTGTCCCAGTTACCAGGCTGTACGCATGGGGTAGC
	D1_FIP_ind4	GCTGCGTTGTGTCTTGGGAGGTTCGGATTCTATCGTGTTTCCCTACTGTACGCATGGGGTAGC
	D1_FIP_ind5	GCTGCGTTGTGTCTTGGGAGGCTTGTCCAGGGTTTGTGTAACCTTCTGTACGCATGGGGTAGC
	D1_FIP_ind6	GCTGCGTTGTGTCTTGGGAGGTTCTCGCAAAGGCAGAAAGTAGTCCTGTACGCATGGGGTAGC
	D1_FIP_ind7	GCTGCGTTGTGTCTTGGGAGGGTGTTACCGTGGGAATGAATCCTTCTGTACGCATGGGGTAGC
	D1_FIP_ind8	GCTGCGTTGTGTCTTGGGAGGTTCAGGGAACAAACCAAGTTACGTCTGTACGCATGGGGTAGC
	D1_FIP_ind9	GCTGCGTTGTGTCTTGGGAGGAACTAGGCACAGCGAGTCTTGGTTCTGTACGCATGGGGTAGC
	D1_FIP_ind10	GCTGCGTTGTGTCTTGGGAGGAAGCGTTGAAACCTTTGTCCTCTCCTGTACGCATGGGGTAGC
for type 2	D2_FIP_ind1	TTGGGCCCCCATTGTTGCTGAAGAAAGTTGTCGGTGTCTTTGTGAGTGGACTAGCGGTTAGAGG
	D2_FIP_ind2	TTGGGCCCCCATTGTTGCTGTCGATTCCGTTTGTAGTCGTCTGTAGTGGACTAGCGGTTAGAGG
	D2_FIP_ind3	TTGGGCCCCCATTGTTGCTGGAGTCTTGTGTCCCAGTTACCAGGAGTGGACTAGCGGTTAGAGG
	D2_FIP_ind4	TTGGGCCCCCATTGTTGCTGTTCGGATTCTATCGTGTTTCCCTAAGTGGACTAGCGGTTAGAGG
	D2_FIP_ind5	TTGGGCCCCCATTGTTGCTGCTTGTCCAGGGTTTGTGTAACCTTAGTGGACTAGCGGTTAGAGG
	D2_FIP_ind6	TTGGGCCCCCATTGTTGCTGTTCTCGCAAAGGCAGAAAGTAGTCAGTGGACTAGCGGTTAGAGG
	D2_FIP_ind7	TTGGGCCCCCATTGTTGCTGGTGTTACCGTGGGAATGAATCCTTAGTGGACTAGCGGTTAGAGG
	D2_FIP_ind8	TTGGGCCCCCATTGTTGCTGTTCAGGGAACAAACCAAGTTACGTAGTGGACTAGCGGTTAGAGG
	D2_FIP_ind9	TTGGGCCCCCATTGTTGCTGAACTAGGCACAGCGAGTCTTGGTTAGTGGACTAGCGGTTAGAGG
	D2_FIP_ind10	TTGGGCCCCCATTGTTGCTGAAGCGTTGAAACCTTTGTCCTCTCAGTGGACTAGCGGTTAGAGG
for type 3	D3_FIP_ind1	TGGCTTTTGGGCCTGACTTCAAGAAAGTTGTCGGTGTCTTTGTGGAAGAAGCTGTGCAGCCTG
	D3_FIP_ind2	TGGCTTTTGGGCCTGACTTCTCGATTCCGTTTGTAGTCGTCTGTGAAGAAGCTGTGCAGCCTG
	D3_FIP_ind3	TGGCTTTTGGGCCTGACTTCGAGTCTTGTGTCCCAGTTACCAGGGAAGAAGCTGTGCAGCCTG
	D3_FIP_ind4	TGGCTTTTGGGCCTGACTTCTTCGGATTCTATCGTGTTTCCCTAGAAGAAGCTGTGCAGCCTG
	D3_FIP_ind5	TGGCTTTTGGGCCTGACTTCCTTGTCCAGGGTTTGTGTAACCTTGAAGAAGCTGTGCAGCCTG
	D3_FIP_ind6	TGGCTTTTGGGCCTGACTTCTTCTCGCAAAGGCAGAAAGTAGTCGAAGAAGCTGTGCAGCCTG
	D3_FIP_ind7	TGGCTTTTGGGCCTGACTTCGTGTTACCGTGGGAATGAATCCTTGAAGAAGCTGTGCAGCCTG
	D3_FIP_ind8	TGGCTTTTGGGCCTGACTTCTTCAGGGAACAAACCAAGTTACGTGAAGAAGCTGTGCAGCCTG
	D3_FIP_ind9	TGGCTTTTGGGCCTGACTTCAACTAGGCACAGCGAGTCTTGGTTGAAGAAGCTGTGCAGCCTG
	D3_FIP_ind10	TGGCTTTTGGGCCTGACTTCAAGCGTTGAAACCTTTGTCCTCTCGAAGAAGCTGTGCAGCCTG
for type 4	D4_FIP_ind1	TGGGAATTATAACGCCTCCCGAAGAAAGTTGTCGGTGTCTTTGTGCCACGGCTTGAGCAAACC
	D4_FIP_ind2	TGGGAATTATAACGCCTCCCGTCGATTCCGTTTGTAGTCGTCTGTCCACGGCTTGAGCAAACC
	D4_FIP_ind3	TGGGAATTATAACGCCTCCCGGAGTCTTGTGTCCCAGTTACCAGGCCACGGCTTGAGCAAACC
	D4_FIP_ind4	TGGGAATTATAACGCCTCCCGTTCGGATTCTATCGTGTTTCCCTACCACGGCTTGAGCAAACC
	D4_FIP_ind5	TGGGAATTATAACGCCTCCCGCTTGTCCAGGGTTTGTGTAACCTTCCACGGCTTGAGCAAACC
	D4_FIP_ind6	TGGGAATTATAACGCCTCCCGTTCTCGCAAAGGCAGAAAGTAGTCCCACGGCTTGAGCAAACC
	D4_FIP_ind7	TGGGAATTATAACGCCTCCCGGTGTTACCGTGGGAATGAATCCTTCCACGGCTTGAGCAAACC
	D4_FIP_ind8	TGGGAATTATAACGCCTCCCGTTCAGGGAACAAACCAAGTTACGTCCACGGCTTGAGCAAACC
	D4_FIP_ind9	TGGGAATTATAACGCCTCCCGAACTAGGCACAGCGAGTCTTGGTTCCACGGCTTGAGCAAACC
	D4_FIP_ind10	TGGGAATTATAACGCCTCCCGAAGCGTTGAAACCTTTGTCCTCTCCCACGGCTTGAGCAAACC

c

for type 1	D1S	GGACTGCGTATGGAGTTTTG
	D1C	ATGGGTTGTGGCCTAATCAT
for type 2	D2Sv3	AGRTTYGTCTGCAAACACTCC
	D2Cv3	GTGTTACTTTRATTTCCTTG
for type 3	D3S	GTGCTTACACAGCCCTATTT
	D3C	TCCATTCTCCCAAGCGCCTG
for type 4	D4S	CCATTATGGCTGTGTTGTTT
	D4C	CTTCATCCTGCTTCACTTCT

Table S6 | Primers used for the LAMP amplification.
Primers without barcoding oligos (a), primers containing barcoding oligos for multiplexing (b), primers for RT-PCR validation (c).

a

Run ID	# 2D total reads	# total assigned reads	% assigned	Index 1	Index 2	Index 3	Index 4	Index 5	Index 6	Index 7	Index 8	Index 9	Index 10
1000	3,965	2,597	65%	ND	334	329	328	70	221	167	397	332	419
1001	927	587	63%	72	69	67	105	ND	72	40	33	19	110
1002	2,777	1,773	64%	258	484	144	145	44	204	120	0	159	215
1003	2,599	1,541	59%	82	215	87	115	87	270	204	50	110	321
1004	440	280	64%	69	ND	5	30	31	24	33	27	23	38
1005	1,480	846	57%	182	ND	117	71	102	50	118	0	72	134
1006	596	311	52%	9	12	30	53	16	39	52	16	28	56
1007	1,673	847	51%	ND	52	57	269	123	201	50	31	61	3
1008	1,503	819	54%	ND	134	99	247	ND	114	66	78	ND	81
1009	3,631	1,824	50%	405	56	ND	70	31	27	33	29	498	675
1010	710	337	47%	93	5	5	5	7	17	5	37	90	73
1011	1,321	784	59%	175	11	1	13	10	7	222	0	158	187
1012	1,161	746	64%	201	13	ND	10	13	12	18	8	245	226
1013	3,641	2,206	61%	593	66	ND	46	24	29	40	ND	731	677
1014	1,668	891	53%	341	21	ND	2	17	21	13	0	451	25
1015	1,768	1,138	64%	683	18	ND	37	35	51	27	0	239	48
1016	1,830	1,383	76%	517	81	ND	37	ND	52	53	255	388	ND
1017	2,525	1,569	62%	364	ND	ND	83	84	98	46	125	486	283
1018	4,186	2,731	65%	133	177	91	115	390	402	491	59	277	596
1019	7,815	6,196	79%	439	203	ND	1309	620	446	345	548	525	1761
1020	4,486	2,842	63%	1181	199	ND	151	827	211	125	148	ND	ND
1021	7,854	4,248	54%	814	674	ND	1056	486	439	739	40	ND	ND
1022	1,118	628	56%	45	178	ND	61	66	87	47	35	109	ND
1023	3,850	2,794	73%	ND	660	100	58	124	465	520	455	383	29
1024	4,287	3,173	74%	69	423	4	126	82	251	713	607	407	491
1025	3,327	2,453	74%	222	424	197	310	70	79	305	374	425	47
1026	2,027	1,473	73%	32	199	102	19	148	215	184	279	295	ND
1027	936	627	67%	8	18	112	51	34	209	46	149	ND	ND

b

Mutation	Sample ID detected by MinION	Sample ID detected by Illumina
Den1:10581:C:G	2012-14_045, 2012-14_059, 2012-14_060, 2012-14_068, 2012-14_243, 2012-14_252, 2015_B15, 2015_B48, students_08, students_09, students_18	2012-14_059, 2012-14_060, 2012-14_243, 2015_B15, students_08, students_09, 2012-14_022, 2012-14_025, 2012-14_026, 2012-14_034, 2012-14_043, 2012-14_053, 2012-14_066, 2012-14_105, 2012-14_109, 2012-14_125, 2012-14_135, 2012-14_189, 2012-14_239, 2015_B16, 2015_B50, students_11, students_17, 2012-14_024, 2012-14_132, 2012-14_165, 2012-14_179
Den2:10553:C:T	2015_B18, 2012-14_166, 2015_B50, 2012-14_146, 2012-14_170	2015_B50, 2012-14_146, 2012-14_004
Den2:10555:A:G	2015_B18, 2012-14_166, 2015_B50, 2012-14_146, 2012-14_170, 2012-14_004	2012-14_146, 2012-14_004
Den2:10559:G:C	2015_B18	
Den2:10560:A:G	2012-14_166, 2015_B50	2015_B50
Den2:10573:G:A	2015_B18, 2012-14_166, 2015_B50, 2012-14_146, 2012-14_170, 2012-14_004	2015_B50, 2012-14_146, 2012-14_004
Den3:10357:C:T	2012-14_009	
Den3:10391:T:C	2015_B30, 2015_B36, 2015_B37	2015_B50
Den3:10395:A:G	2012-14_145	2012-14_145
Den3:10431:G:A	2015_B37	
Den3:10436:A:G	2012-14_005, 2012-14_008, 2012-14_017, 2012-14_018, 2012-14_033, 2012-14_048, 2012-14_137, 2012-14_138, 2012-14_146, 2012-14_147, 2012-14_149, 2012-14_152, 2012-14_162, 2012-14_170, 2015_B56, 2012-14_009, 2012-14_145	2012-14_005, 2012-14_008, 2012-14_017, 2012-14_048, 2012-14_146, 2012-14_147, 2012-14_149, 2012-14_152, 2012-14_009, 2012-14_145
Den3:10466:A:T		2012-14_147
Den4:10378:G:T		2012-14_004, 2012-14_149, 2012-14_150, 2012-14_184
Den4:10387:C:T	2012-14_167	
Den4:10398:C:T	2015_B18	
Den4:10399:T:G	2012-14_167	
Den4:10403:C:A		2012-14_034
Den4:10409:G:A	2012-14_151	

Den4:10413:A:C	2012-14_040
Den4:10472:C:G	2012-14_104

c

Mutation	Sample ID detected by MinION	Sample ID detected by Illumina
DENV1:10527:C:T	13S03799, 13S03834	
DENV1:10581:C:G	13S03834, 13S03857	13S03732, 13S03740, 13S03799, 13S03801, 13S03806, 13S03857
DENV2:10553:C:T	13S03873	
DENV2:10555:A:G	13S03873	
DENV2:10570:C:T	13S03873	
DENV2:10573:G:A	13S03873	
DENV3:10403:G:C		13S03866
DENV3:10436:A:G	13S03734, 13S03746, 13S03763, 13S03764, 13S03770, 13S03776, 13S03789, 13S03792, 13S03818, 13S03827, 13S03843, 13S03855, 13S03866	13S03866, 13S03746, 13S03763, 13S03764, 13S03770, 13S03776, 13S03780, 13S03789, 13S03792, 13S03810, 13S03818, 13S03827, 13S03836, 13S03843, 13S03855, 13S03865, 13S03872
DENV4:10388:G:T	13S03748, 13S03754, 13S03756, 13S03781, 13S03825	13S03748, 13S03754, 13S03756, 13S03766, 13S03795, 13S03820, 13S03833, 13S03845, 13S03846, 13S03854, 13S03868, 13S03825, 13S03859
DENV4:10405:C:T		13S03825, 13S03859

d

Mutation	Sample ID detected by MinION	Sample ID detected by Illumina
DENV1:10527:C:T	01-0777	01-0777, 02-0083
DENV1:10581:C:G	02-0083	01-0777, 02-0083
DENV2:10553:C:T	02-0074	02-0074
DENV2:10555:A:G	02-0074	02-0074
DENV2:10568:G:A	02-0074, 03-0067, 03-0164	02-0074, 03-0067, 03-0164
DENV2:10571:T:C	03-0164	03-0164
DENV2:10573:G:A	02-0074	
DENV2:10577:G:C	02-0074	02-0074
DENV2:10578:A:G		02-0074
DENV3:10436:A:G	02-0163	
DENV4:10388:G:T	01-0122, 03-0031	01-0122, 03-0031

Table S7 | Results of MinION analysis by the multiplex analysis for clinical samples.
a, The number of sequences associated with the indicated barcoding oligos. **b**, **c, d,** SNVs detected from multiplexed MinION reads. The positions and patterns of the SNVs and their validation analyses are shown for the indicated samples from Indonesia (**b**), Vietnam (**c**), and Thailand (**d**).

calculation in this paper: identical base / (identical base + mismatch base + insertion base + deletion base)

	Den1	Den2	Den3	Den4
last tuned (this paper)	76.9%	78.6%	81.7%	78.5%
last460	76.9%	78.1%	80.2%	77.1%
last460 (MarginAlign)	72.3%	71.9%	73.4%	71.2%
bwa	77.6%	78.2%	80.4%	78.4%
bwa (MarginAlign)	75.1%	74.9%	78.4%	76.4%

calculation (according to MarginStats): identical base / (identical base + mismatch base + insertion base)

	Den1	Den2	Den3	Den4
last tuned (this paper)	84.8%	83.9%	85.7%	82.4%
last460	84.9%	83.8%	84.7%	81.2%
last460 (MarginAlign)	82.5%	80.3%	80.0%	76.0%
bwa	85.4%	83.8%	84.9%	82.3%
bwa (MarginAlign)	86.1%	83.6%	85.1%	82.0%

<table>
<tr><th>Tool</th><th>URL</th><th>Version</th><th>Command</th></tr>
<tr><td>last (this paper)</td><td>http://last.cbrc.jp/</td><td>548(658)</td><td>lastal -q12 -a15 -b3 -e150 -m100 -Q1 -j4 $indexFile $fastq| last-split -m1 -
maf-convert.py sam -d $mafFile | samtools view -Sb - | samtools sort - -o bam</td></tr>
<tr><td>last460</td><td>http://last.cbrc.jp/</td><td>460</td><td>lastal -s 2 -T 0 -Q 0 -a 1 $indexFile $fasta > $mafFile
maf-convert.py sam -d $mafFile | samtools view -Sb - | samtools sort - -o bam</td></tr>
<tr><td>bwa</td><td>http://bio-bwa.sourceforge.net/</td><td>0.7.12-r1044</td><td>bwa mem -x ont2d $ref $fastq | samtools view -Sb - | samtools sort - -o bam</td></tr>
<tr><td>marginAlign</td><td>https://github.com/benedictpaten/marginAlign/</td><td>0.1</td><td>marginAlign ..$fastq .$refFasta $sam --em (--bwa) --outputModel $model --jobTree $treeoutputdir</td></tr>
</table>

Table S8 | MinION performance between the pipeline described in this paper and MarginAlign.

Category	#of samples	Diagnosed serotypes # (%)					
		Serotype detected	%	Serotype elusive	%	ND	%
NS1+	67	49	73%	6	9%	12	18%
Ig+	60	40	67%	6	10%	14	23%
No evidence	14	2	14%	0	0%	12	86%
Total	141	91	65%	12	8%	38	27%

Table S9 | Serotyping results and medical evidence

Year	Region	Total samples	Target genes	Serotype	Genotype	Amino Acid	Reference
2004	Malaysia	101	E protein	DENV1	Ic (98%)<br><br>II (2%)	D37, K120, E157<br>D37, T88, D157, K120, T225, S339, V345	Boon-Teong Teoh (2013)
1974-2001	Thailand	105	E protein	DENV2	Asian I (96.2%)<br>Asian/American (3.8%)		Chunlin Zhang (2006)
2003-2008	Vietnam	187	E protein	DENV2	Asian I (74.3%)<br><br>Asian/American (24.6%)	K83, N203, K226, E228, Y346<br>N83, D203, T226, G228, H346	Vu Thi Ty Hang (2010)
2004	Jakarta	10	E protein	DENV3	I	V68, V169, K231, S301, A303, V479	Chwan-Chuen King (2008)
1998	Taiwan	4	E protein	DENV3	II	P124, Y132, D154, V160, V169, N270, V479	Chwan-Chuen King (2008)
1994	Thailand	2	E protein	DENV3	II	V68, P124, D154, V160, N270, V479	Chwan-Chuen King (2008)
2001	Malaysia	5	E protein	DENV4	IIA	V108, L120, I335, D384, V455, L461	Sazaly AbuBakar (2002)
1998-2002	Bangkok	53	E protein	DENV4	I (90.5%)<br>III (9.5%)		Chonticha Klungthong (2004)

Table S10 | Genotyping of each DENV serotype

Flow cell version	Price	Number of experiment	Cost per experiment
FLO_MAP002	$1,998.00	2	$999.00
FLO_MAP003	$1,998.00	2	$999.00
FLO_MAP103	$900.00	1	$900.00
FLO_MAP103.48	$24,000.00	48	$500.00
FLO_MAP104	$900.00	1	$900.00
FLO_MAP104.48	$24,000.00	48	$500.00

Reagent version	Price	Number of experiment	Cost per experiment
SQK_MAP004	$499.00	3	$166.33
SQK_MAP005	$599.00	6	$99.83
SQK_MAP006	$599.00	6	$99.83
SQK_MAP007	$599.00	6	$99.83

Table S11 | Price of MinION flow cell and reagents

Method S1 | RT-PCR protocol

SuperScript® One-Step RT-PCR System with Platinum® Taq DNA Polymerase
https://www.thermofisher.com/order/catalog/product/10928042?ICID=search-product

2X Reaction Mix	25 µL
Template RNA	1 µL
Sense Primer (10 µM)	1 µL
Anti-sense Primer (10 µM)	1 µL
RT/ Platinum® Taq Mix	0.5 µL
Autoclaved distilled water	6.5 µL

Perform 1 cycle of:

- 50°C for 30 minutes
- 94°C for 2 minutes

Perform 40 cycles of:

- Denature: 94°C for 30 seconds
- Annealing: 53°C for 30 seconds
- Extension: 72°C for 1 minute/kb

Perform 1 cycle of 72ºC for 2 minutes
